# Cost and cost-effectiveness of tuberculosis treatment shortening: a model-based analysis

**DOI:** 10.1186/s12879-016-2064-3

**Published:** 2016-12-01

**Authors:** G. B. Gomez, D. W. Dowdy, M. L. Bastos, A. Zwerling, S. Sweeney, N. Foster, A. Trajman, M. A. Islam, S. Kapiga, E. Sinanovic, G. M. Knight, R. G. White, W. A. Wells, F. G. Cobelens, A. Vassall

**Affiliations:** 1 Amsterdam Institute for Global Health and Development and Department of Global Health, Academic Medical Center, University of Amsterdam, Trinity Building C, Pietersbergweg 17, Amsterdam, 1105 BM The Netherlands; 2 Department of Global Health and Development, London School of Hygiene and Tropical Medicine, London, UK; 3 Department of Epidemiology, Johns Hopkins Bloomberg School of Public Health, Baltimore, USA; 4 Federal University of Rio de Janeiro, Rio de Janeiro, Brazil; 5 Tuberculosis Scientific League, Rio de Janeiro, Brazil; 6 Health Economics Unit, School of Public Health & Family Medicine, University of Cape Town, Cape Town, South Africa; 7 McGill University, Montreal, Canada; 8 BRAC Health Nutrition and Population Programme, BRAC Centre, Dhaka, Bangladesh; 9 Mwanza Intervention Trials Unit, National Institute for Medical Research, Mwanza, Tanzania; 10 TB Modelling Group, Department of Infectious Disease Epidemiology, London School of Hygiene and Tropical Medicine, London, UK; 11 Global Alliance for TB Drug Development, New York, USA; 12 KNCV Tuberculosis Foundation, The Hague, Netherlands; 13 Present address: United States Agency for International Development, Washington, DC USA

**Keywords:** Tuberculosis, Cost-effectiveness, Economic evaluation, New technologies

## Abstract

**Background:**

Despite improvements in treatment success rates for tuberculosis (TB), current six-month regimen duration remains a challenge for many National TB Programmes, health systems, and patients. There is increasing investment in the development of shortened regimens with a number of candidates in phase 3 trials.

**Methods:**

We developed an individual-based decision analytic model to assess the cost-effectiveness of a hypothetical four-month regimen for first-line treatment of TB, assuming non-inferiority to current regimens of six-month duration. The model was populated using extensive, empirically-collected data to estimate the economic impact on both health systems and patients of regimen shortening for first-line TB treatment in South Africa, Brazil, Bangladesh, and Tanzania. We explicitly considered ‘real world’ constraints such as sub-optimal guideline adherence.

**Results:**

From a societal perspective, a shortened regimen, priced at USD1 per day, could be a cost-saving option in South Africa, Brazil, and Tanzania, but would not be cost-effective in Bangladesh when compared to one gross domestic product (GDP) per capita. Incorporating ‘real world’ constraints reduces cost-effectiveness. Patient-incurred costs could be reduced in all settings. From a health service perspective, increased drug costs need to be balanced against decreased delivery costs. The new regimen would remain a cost-effective option, when compared to each countries’ GDP per capita, even if new drugs cost up to USD7.5 and USD53.8 per day in South Africa and Brazil; this threshold was above USD1 in Tanzania and under USD1 in Bangladesh.

**Conclusion:**

Reducing the duration of first-line TB treatment has the potential for substantial economic gains from a patient perspective. The potential economic gains for health services may also be important, but will be context-specific and dependent on the appropriate pricing of any new regimen.

**Electronic supplementary material:**

The online version of this article (doi:10.1186/s12879-016-2064-3) contains supplementary material, which is available to authorized users.

## Background

Globally, despite advances in diagnostic technologies and treatment success rates, tuberculosis (TB) remains a substantial health problem [1]. A major challenge faced by National TB Programs and patients is the length and complexity of existing regimens. New shortened regimens have potential advantages: improving outcomes through increasing adherence; decreasing time to cure; reducing costs incurred by patients; and reducing treatment delivery costs incurred by health systems [2, 3]. These potential health and economic gains have motivated increasing investments in new regimens in recent years [4–6]. Although recent trials of new TB regimens, such as four-month moxifloxacin-based regimens, have so far proved unsuccessful [4], larger-scale trials including new drugs are ongoing [7]. Understanding the potential benefits and costs of introducing new TB regimens in different epidemiological and health system contexts is therefore critical in the context of the post-2015 global TB Targets.

Previous efforts to quantify the economic and health impact of shortened regimens have been limited; and these efforts have focused on general analyses that are not specific to any particular setting. While such studies can identify key drivers of cost-effectiveness at a general level, further work is required to characterize the cost-effectiveness of new TB regimens at country level [8–11]. Importantly, none of the previous studies were parameterized with locally-collected data; or reflect the influence of health system constraints. Incorporating sound empirical data on health service costs is critical, given that one of the central aims of introducing new shortened first-line regimens is to achieve a reduction in health system burden from TB treatment. Moreover, while previous studies have included estimates of provider costs, none of these studies have considered the potential cost savings to patients. The potential benefits in terms of patient costs are important in the light of the post-2015 global TB target of ensuring that no-one suffers catastrophic expenditures from TB, in the context of Universal Health Coverage.

We explore the cost and cost-effectiveness of introducing a new shortened TB regimen in four countries. We use primary data on both patient and provider incurred costs; and incorporate data on local patterns of TB treatment delivery in our estimates. The aim is to guide further development and in-country adoption of shortened first-line TB regimens.

## Methods

### Model

We used a decision analytic model to compare the cost-effectiveness of a hypothetical four-month regimen to the existing standard of care, a six-month regimen for first-line treatment of TB. Our primary outcomes were the incremental cost per disability-adjusted life-year (DALY) averted, and incremental average total, health service and patient incurred costs. Our population was a cohort 10,000 individuals with newly diagnosed pulmonary TB and no previous treatment history, characterised for four settings, South Africa, Bangladesh, Brazil and Tanzania (Additional file 1: Table S1). The model defined each individual patient in the cohort by smear status (positive or negative), HIV (positive or negative), antiretroviral treatment (ART) (treated or not treated), and resistance profile (pan-sensitive or resistant to at least rifampicin) to reflect the multi-drug resistant TB (MDR-TB) burden in each country.

The treatment approach was informed by national guidelines, which are laid out in table S2 (Additional file 1). At the end of each month, patients could either continue on or complete treatment, be lost to follow-up (default), die, or (in the final month of treatment) fail. Loss to follow-up is defined as a patient whose treatment is interrupted for two consecutive months [12]. After loss to follow-up, we assumed patients may re-enter care, thereafter classified as previously treated. The duration of treatment before default determined the treatment algorithm to be followed once the patient returned to care. The probability of cure after default also depended on the duration of treatment completed before defaulting as per the literature of shorter-course regimens [13]. Patients stopping treatment in the first two months were assumed to receive no benefit from the treatment and have a probability of cure equivalent to the probability of spontaneous recovery. Thereafter, patients stopping treatment received partial benefit from the treatment and have a probability of cure proportional to the length of treatment completed [13].

Treatment failure was defined as a smear positive result at five months or later during treatment [12]. After treatment failure, we assumed that patients complete either a second round of treatment (standard first line in South Africa and Brazil or one course of retreatment in Bangladesh and Tanzania, where retreatment regimens are available) or, where they have been diagnosed as rifampicin-resistant after drug susceptibility testing (DST), one course of MDR-TB treatment. If they failed the second round of treatment, they were considered not to receive additional treatment, and become chronic TB cases until death. Cases of relapse (a new episode of TB after a period of no TB) were excluded [12].

The model was built using TreeAge software. Several schematics of the model are presented in the Additional file 1: Figure S1 to Figure S3, while the main parameter values used are presented in Table 1.

**Table 1:** Parameters

Parameter, value [range]	South Africa	Brazil	Bangladesh	Tanzania	Reference
Population distribution					
Smear-positivity: HIV-negative, HIV-positive no ART, HIV-positive ART	0.69, 0.35, 0.45				[29] [30]
MDR prevalence, among new patients	1.8%	1.4%	1.4%	1.1%	[1, 31, 32]
Prevalence of HIV in TB patients	62%	17%	3%	37%	[1, 33, 34]
Diagnosis of TB					
TB diagnosis, sensitivity, smear, HIV negative	0.72 [0.62–0.82]				[29, 35]
TB diagnosis, sensitivity, smear, HIV positive	0.47 [0.51–0.43]				[29, 35]
TB diagnosis, sensitivity, GeneXpert, smear positive	0.98 [0.97–0.99]				[36]
TB diagnosis, sensitivity, GeneXpert, smear negative	0.68 [0.59–0.75]				[36]
TB diagnosis, specificity, GeneXpert, all	0.98 [0.97–0.99]				[36]
TB diagnosis, specificity, smear, all	1				assumption
RIF-resistance diagnosis, sensitivity, GeneXpert, all	0.94 [0.87–0.97]				[36]
RIF-resistance diagnosis, specificity, GeneXpert, all	0.98 [0.97–0.99]				[36]
Outcomes (first line treatment)					
Pr mortality:					
Pan-sensitive, HIV negative	0.03 [0.02–0.03]				[37]
MDR, HIV negative	0.11 [0.08–0.13]				[37]
Pan-sensitive, HIV positive, ART	0.07 [0.05–0.09]				[38]
Pan-sensitive, HIV positive, no ART	0.33 [0.30–0.43]				[38]
MDR, HIV positive, ART	0.11 [0.10–0.21]				[38, 39]
MDR, HIV positive, no ART	0.85 [0.72–0.98]				[39]
Pr cure, if treatment completed:					
Pan-sensitive	0.97 [0.95–0.98]				[13]
MDR	0.50 [0.40–0.55]				[37]
Pr cure, if less than 2 months treatment completed					
HIV negative, smear negative	0.20 [0.15–0.25]				[40–42]
HIV negative, smear positive	0.30 [0.20–0.40]				[40–42]
HIV positive, smear neg/pos, no ART	0 [0–0.05]				[40–42]
HIV positive, smear negative, ART	0.10 [0.05–0.15]				[40–42]
HIV positive, smear positive, ART	0.05 [0–0.10]				[40–42]
Pr cure, if default at (standard 6mo regimen):					
2–3 months, pan-sensitive	0.68 [0.50–0.80]				[43]
2–3 months, MDR	0.35 [0.21–0.45]				[43]
4–5 months, pan-sensitive	0.86 [0.70–0.89]				[44, 45]
4–5 months, MDR	0.48 [0.29–0.51]				[44, 45]
Pr cure, if default at (new 4mo regimen):					
2–3 months, pan-sensitive	0.74 [0.57–0.83]				Assumption
2–3 months, MDR	0.38 [0.23–0.47]				Assumption
Outcomes (second round of treatment)					
Pr patients returning to care after default	0.21 [0.10–0.70]				[46]
Pr patients staying in care after failure	0.60 [0.40–0.80]				Assumption
Pr mortality					
HIV negative/positive ART, pan-sensitive	0.06 [0.04–0.07]				[37]
HIV negative/positive ART, MDR	0.15 [0.10–0.20]				[37]
HIV positive no ART, pan-sensitive	0.33 [0.30–0.43]				[38]
HIV positive no ART, MDR	0.85 [0.72–0.98]				[39]
MDR treatment and long term outcomes					
Pr cure, MDR treatment (including default)	0.65–0.80				[47]
Mortality during MDR treatment	0.10				[48]
Pr long term mortality (chronic TB patient or default patient if no return to care)	0.75 [0.50–0.99]				[48]
Self-cure among chronic TB patients	0.01				[48]
DALYs averted (discounted at 0.03/year)					
HIV negative, smear negative	12.5 [11.3–13.8]	19.3 [17.4–21.3]	15.3 [13.7–16.8]	14.2 [12.8–15.7]	Additional file 1
HIV negative, smear positive	15.2 [13.7–16.7]	22.0 [19.8–24.2]	17.9 [16.1–19.7]	16.9 [15.2–18.6]	Additional file 1
HIV positive, smear negative, no ART	1.8 [1.6–2.0]	1.8 [1.6–2.0]	1.8 [1.6–2.0]	1.8 [1.6–2.0]	Additional file 1
HIV positive, smear positive, no ART	2.0 [1.8–2.2]	2.0 [1.8–2.2]	2.0 [1.8–2.2]	2.0 [1.8–2.2]	Additional file 1
HIV positive, smear negative, ART	9.9 [8.9–10.9]	9.9 [8.9–10.9]	9.9 [8.9–10.9]	9.9 [8.9–10.9]	Additional file 1
HIV positive, smear positive, ART	10.1 [9.1–11.1]	10.1 [9.1–11.1]	10.1 [9.1–11.1]	10.1 [9.1–11.1]	Additional file 1

*HIV* human immunodeficiency virus, *TB* tuberculosis, *ART* antiretroviral treatment, *MDR* multidrug resistant, *Pr* probability

Parameters with one value are included in the model as point estimates; parameters with a value and a range were included in the model as triangular distributions; parameters with only a range of two values were included in the model as a uniform distribution

### Intervention

We modelled a non-inferior four-month new regimen compared to the current six-month regimen, on the assumption that non-inferiority would be the minimal aim of new trials. Non-inferiority refers to the efficacy of the regimen. Given this conservative assumption, the potential impact of the new shortened regimen on health outcomes results from an increase in effectiveness due to a higher probability of patients completing a shortened treatment regimen and a higher probability of cure if patients stop treatment early after taking at least two months of the shortened regimen. Introduction of the new regimen into practice was set to follow national TB treatment guidelines with regards to DST algorithms, eligibility for first-line therapy, monitoring, standardised MDR treatment, directly observed therapy (DOT), and ART eligibility for HIV/TB patients (Additional file 1: Table S3). Where DST was available, we assumed that a case resistant to rifampicin will not be eligible for either the new or current first-line treatment regimen.

### Costs

Costs related to TB treatment were estimated from a societal perspective in South Africa, Bangladesh, Brazil, and Tanzania, using extensive primary costing surveys. All cost estimates are presented in 2013 USD [14, 15]. Summary costs are presented in Table 2; full details on the costing methodology can be found in publications elsewhere [3, 16–19] and summarized in table S4, while detailed unit costs are presented in Additional file 1: Table S5.

**Table 2:** Costs

	South Africa	Brazil	Bangladesh	Tanzania	Reference
a. Guidelines					
Healthcare provider costs					
First-line treatment, IP, 1mo (excl drugs)	200 (152–230)	333 (117–479)	17 (12–21)	65 (24–106)	[16, 18, 19]
First-line treatment, CP, 1mo (excl drugs)	54 (41–62)	333 (117–479)	11 (7–15)	16 (7–24)	[16, 18, 19]
Drugs, first-line, IP, 1mo	16	7 (6–9)	7 (6–8)	6	[16, 18–20]
Drugs, first-line, CP, 1mo	19	4 (3–6)	3 (3–3)	2	[16, 18–20]
Retreatment: all	n/a	n/a	213 (160–266)	430 (310–549)	[16, 18, 19]
MDR treatment: all	10,215 (8,619–24,580)	5,223 (4,800–5,348)	4,262 (3,836–4,688)	2,507 (2,454–2,561)	[16, 18, 19]
ART cost in year 1	1,128 (1,117–1,139)	5,875 (5,288–6,463)^a^	800 (720–880)	315 (283–346)	[21, 22, 49–52]
ART cost per year (after year 1)	639 (575–703)		600 (540–660)	277 (249–304)	[21, 22, 49–52]
Patient costs					
First-line treatment, IP, 1mo	149 (87–164)	40 (8–131)	314 (283–346)	186 (167–204)	[3, 16, 17]
First-line treatment, CP, 1mo	117 (34–129)	40 (8–131)	31 (28–34)	44 (40–48)	[3, 16, 17]
Retreatment: all	n/a	n/a	135 (121–148)	354 (319–390)	[3, 16, 17]
MDR treatment: all	3,319 (2,987–3,650)	280 (102–1142)	213 (192–234)	454 (409–499)	[3, 16, 17]
ART cost in year 1	106 (96–117)	23 (4–43)	8 (7–8)	24 (22–26)	[53]
ART cost per year (after year 1)	85 (77–93)	9 (2–17)	3 (3–3)	10 (9–11)	[53]
Cost per visit to healthcare facility	8 (7–9)	5 (1–9)	2 (1–2)	5 (4–5)	[3, 16, 17]
b. Current					
Healthcare provider costs					
First-line treatment, IP, 1mo (excl drugs)	61 (40–96)	133 (59–285)	17 (12–21)	35 (24–45)	[16, 18, 19]
First-line treatment, CP, 1mo (excl drugs)	16 (11–26)	133 (59–285)	11 (8–15)	16 (7–24)	[16, 18, 19]
Drugs, first-line, IP, 1mo	16	7 (6–9)	7 (6–8)	6	[16, 18–20]
Drugs, first-line, IP, 1mo	19	4 (3–6)	3 (3–3)	2	[16, 18–20]
Retreatment: all	n/a	n/a	213 (160–266)	429 (310–549)	[16, 18, 19]
MDR treatment: all	10,215 (8,619–24,580)	5,223 (4,800–5,348)	4,262 (3,836–4,688)	2,507 (2,454–2,561)	[16, 18, 19]
ART cost in year 1	1,128 (1,117–1,139)	5,875 (5,288–6,463)^a^	800 (720–880)	315 (283–346)	[21, 22, 49–52]
ART cost per year (after year 1)	639 (575–703)		600 (540–660)	277 (249–304)	[21, 22, 49–52]
Patient costs					
First-line treatment, IP, 1mo	60 (35–66)	40 (8–131)	314 (283–346)	144 (139–149)	[3, 16, 17]
First-line treatment, CP, 1mo	27 (8–30)	40 (8–131)	31 (28–34)	41 (37–44)	[3, 16, 17]
Retreatment: all	n/a	n/a	135 (121–148)	354 (319–390)	[3, 16, 17]
MDR treatment: all	3,319 (2,987–3,650)	280 (102–1,142)	213 (192–234)	454 (409–499)	[3, 16, 17]
ART cost in year 1	106 (96–117)	23 (4–43)	8 (7–8)	24 (22–26)	[53]
ART cost per year (after year 1)	85 (77–94)	9 (2–17)	3 (3–3)	10 (9–11)	[53]
Cost per visit to healthcare facility	8 (7–9)	5 (1–9)	2 (1–2)	5 (4–5)	[3, 16, 17]

*IP* intensive phase, *CP* continuation phase, *mo* month, *excl* excluding, *ART* antiretroviral treatment, *MDR* multidrug resistant, *DST* drug resistance testing

^a^Average cost per year

#### Health service-related costs

We included service provider costs for all countries and, where feasible, we also included costs incurred above site level, such as monitoring and evaluation and coordination costs. In South Africa, we sourced unit cost data from the “Xpert for TB: Evaluating a New Diagnostic” (XTEND) trial. Data from this trial were collected from eight clinics, 20 laboratories and three MDR treatment sites [18]. In Bangladesh, Tanzania, and Brazil, we conducted health facility costing studies in nine, six, and ten sites respectively. We used a systematic review of previous costing studies to support our estimates, describe parameter bounds and assess representativeness, and, in the case of Tanzania (as we were not able to cost from any community-based DOT activities for feasibility reasons), community-based TB-specific costs were also sourced from the literature [20]. Other costs, such as MDR-TB and ART treatment costs, were sourced from the literature [21–24]. We conducted our primary analysis using a new regimen cost of USD1 per day, in line with previous studies [8].

#### Patient-related costs

Patient data from Bangladesh and Tanzania on direct and indirect costs were sourced from a recent study [3]. In South Africa, XTEND study’s patient cost data were used [17]. In Brazil, we interviewed 126 patients from ten facilities located between the municipality of Rio and peri-urban areas ensuring a mix of wealthier and poorer areas [16]. A review of previous costing studies was used to assess the representativeness and explore uncertainty; and to source information on patient costs for ART in HIV co-infected patients and MDR-TB patients in all countries, except South Africa.

### Analysis

We calculated DALYs averted from patients being cured using the standard formula [25]. Further details on DALY assumptions can be found in Additional file 1: Table S6. We then modelled the intervention using a two-stage approach. Firstly, we modelled the full implementation of the TB treatment guidelines (including full adherence to ART treatment guidelines and MDR treatment coverage) against a locally-parameterised context (epidemiological and study population characteristics). Default rate in this scenario has been set to be at a programmatic minimum of 1–2%, as observed in the recent trial for shortened first-line regimens [4].

In a second stage, we explored the sensitivity of our results to a scenario reflecting current practice, where providers did not adhere to the guidelines and patients have a higher rate of default, reflecting ‘real world’ conditions. Default in this scenario is equal to the one reported by countries in the 2014 Global TB report [1]. We considered non-adherence of two types: non-adherence to the coverage of complementary services (ART and MDR-TB treatment), which affects survival once treatment is completed, and non-adherence to first-line treatment monitoring (directly observed therapy, DOT) by providers. With respect to the former we applied the current coverage levels for ART and MDR-TB treatment; with respect to the latter we allowed the unit costs (and patient costs) of both the standard of care and the new regimen to reflect current levels of DOT. Table 3 shows the coverage levels by scenario.

**Table 3:** Scenario parameters: coverage levels for GeneXpert, MDR treatment and ART in HIV co-infected individuals

	South Africa	Brazil	Bangladesh	Tanzania	reference
All scenarios					
GeneXpert coverage in new patients	100%	100%	0%	0%	assumption
GeneXpert coverage in previously treated	100%	100%	100%	100%	assumption
Guidelines					
ART if HIV/TB	100%	100%	100%	100%	[54–61]
MDR treatment	100%	100%	100%	100%	[56, 59, 60, 62]
Default rate	1–2%	1–2%	1–2%	1–2%	[4]
Current					
ART if HIV/TB	66%	54.7%	100%	73%	[1, 63]
MDR treatment	60%	44.5%	15.4%	12.3%	[1, 64]
Default rate	8% [6–10]	21% [19–23]	2% [1.8–2.2]	3% [2–4]	[1]

*ART* antiretroviral treatment, *HIV* human immunodeficiency virus, *TB* tuberculosis, *MDR* multidrug resistant. Current coverage of ART in HIV/TB co-infection as reported in Global TB report 2014 [1], except for Brazil where this is not reported. For Brazil, we calculated the coverage of ART in HIV/TB co-infection as 84% ART coverage in general HIV patients [63] of 65% TB patients knowing their HIV status. Current coverage of MDR TB treatment was calculated from the Global TB report 2014 [1] from the prevalence of MDR among new and retreatment patients times the number of notifications for new and retreatment patients respectively. This was considered the denominator (total number of MDR patients). The numerator (patients on MDR treatment) was sourced from the Global TB report 2014. For South Africa, we added the estimated current MDR coverage levels as per the National Department of Health [64]. Note: parameters with one value are included in the model as point estimates; parameters with a value and a range were included in the model as triangular distributions; parameters with only a range of two values were included in the model as a uniform distribution

We used a probabilistic sensitivity analysis (Monte Carlo simulation) to randomly sample parameters from distributions (defined in Table 1), conducting 1,000 simulations each of random populations of 10,000 individuals. We report the standard deviations and 95% uncertainty ranges (2.5^th^ and 97.5^th^ percentiles). We explored the main drivers of uncertainty in our estimates in a series of one-way sensitivity analyses by varying each of the following variables over widest plausible ranges sourced from the literature: default rate during treatment, mortality during first-line treatment and after default, prevalence of MDR, no cure if treatment was partially completed, probability of returning to care after default, length of survival if on ART, and discount rate.

Finally, given the uncertainty around drug prices, we conducted a threshold analysis to examine the price for which the incremental cost-effectiveness ratio (ICER) of new TB treatment regimens crosses selected willingness-to-pay (WTP) thresholds. WTP was defined as a quarter, half, or one times the country-specific gross domestic product per capita for the year 2013, to acknowledge possible limited discretionary resources available, and thus effective opportunity costs, within health services in low and middle income countries. Future costs and health gains during the patient’s lifetime were discounted at a rate of 3% per year.

We conducted and present this study following good reporting practices from published standards: the CHEERS statement (Additional file 2: Checklist), and the Bill and Melinda Gates Foundation’s Methods for Economic Evaluation consensus [26, 27].

## Results

**Fig. 1:**
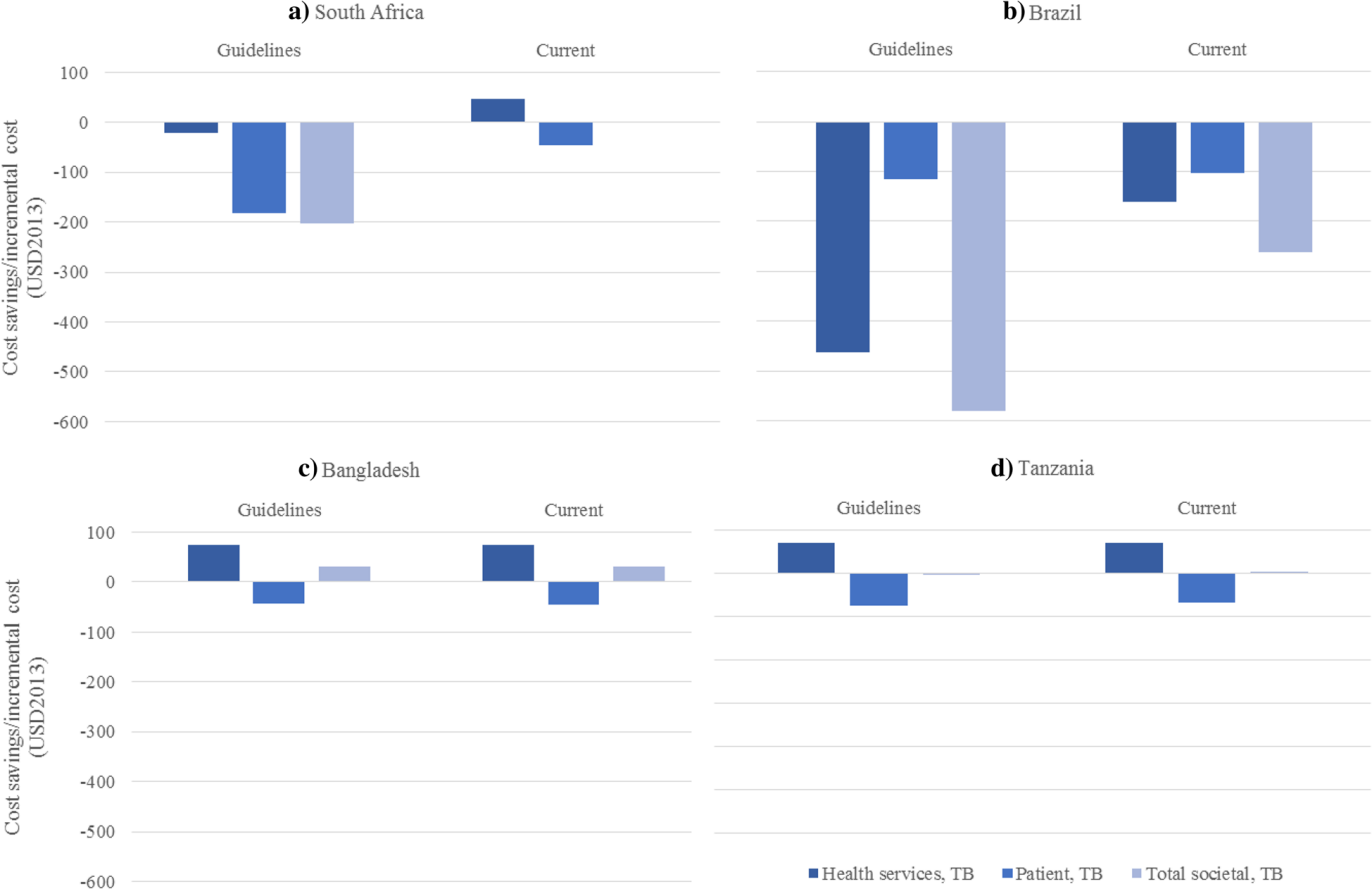
Differences in mean TB-related costs per new TB patient between the new 4 four-month regimen and the six-month regiment, by country, scenario, and payer. Difference of means – negative number refers to cost savings of introducing a shortened regimen compared to baseline (standard treatment). Health services costs are calculated assuming a drug price for the shortened regime of 1USD. These costs do not include ART-related costs. We define the societal perspective as the sum of health service and patient (and their households) perspectives. In the case of South Africa (current scenario) and Tanzania (current and guidelines scenario), the societal perspective costs are very close to cost neutral

Figure 1 shows the cost savings in TB-related costs per new TB patient by country, scenario, and payer. Shortening the treatment regimen from six to four months was projected to reduce patient costs in all settings. These savings are estimated to be highest in Brazil (-30%), then South Africa (-25%) and lowest in Bangladesh (-5%), where the delivery of TB services at the community level minimizes patient costs [3]. From a health service perspective, there is a trade-off between increased drug costs and decreased delivery costs. In settings where existing drug and delivery costs were low, the new regimen (assumed drug price of USD1 per day) increased overall health service costs (USD75 in Bangladesh (60% increase), USD72 in Tanzania, (32% increase)). In contrast, where existing drug and delivery costs are higher, a shorter-course regimen was cost saving (-USD20 in South Africa, 1 · 7% reduction; and -USD463 in Brazil, 24% reduction) (Fig. 1).

When taking into account ‘real world’ provision of TB treatment, these cost savings were substantially lower in South Africa and Brazil. In this case the shortened regimen is no longer cost saving, but results in an 8.4% increase in cost of first-line TB treatment in South Africa, while cost savings are reduced to USD160 in Brazil. Examining the costs related to HIV, we found that the introduction of a new shortened first-line regimen for TB does not substantially affect the incremental costs related to ART (Additional file 1: Table S7).

Although modelling non-inferiority, we observe some benefit for the patient cohort, in terms of mortality reduction and treatment completion (see Additional file 1: Table S8) from a reduction in default. These benefits are larger in high default settings; and in our ‘real world’ analysis. However, this positive effect on mortality and DALYs averted is modest, across all scenarios and countries (Table 4).

**Table 4:** DALYs averted per country and scenario and incremental cost-effectiveness ratios (societal perspective), if drug price is 1USD per day

		Guidelines	Current
South Africa			
Current 6mo Rx	DALY averted, mean (SD)	9.97 (0.23)	8.26 (0.18)
	DALY averted, median (2.5–97.5)	9.97 (9.52–10.42)	8.26 (7.92–8.61)
New 4mo Rx	DALY averted, mean (SD)	10.0 (0.23)	8.37 (0.18)
	DALY averted, median (2.5–97.5)	9.99 (9.55–10.43)	8.37 (8.04–8.72)
	mean difference (%)	0.02 (0.07)	0.11 (0.03)
	ICER, median (2.5–97.5)	CS (CS-26,065)	16.9 (CS-897)
	ICER, calculated mean	CS	13.6
GDP per capita		6,618	
Brazil			
Current 6mo Rx	DALY averted, mean (SD)	16.50 (0.51)	14.68 (0.51)
	DALY averted, median (2.5–97.5)	16.52 (15.54–17.48)	14.67 (13.74–15.65)
New 4mo Rx	DALY averted, mean (SD)	16.54 (0.51)	15.18 (0.51)
	DALY averted, median (2.5–97.5)	16.56 (15.55–17.54)	15.16 (14.25–16.16)
	mean difference (%)	0.04 (0.06)	0.50 (0.08)
	ICER, median (2.5–97.5)	CS (CS-116,251)	CS (CS-362)
	ICER, calculated mean	CS	CS
GDP per capita		11,208	
Bangladesh			
Current 6mo Rx	DALY averted, mean (SD)	16.19 (0.53)	16.17 (0.51)
	DALY averted, median (2.5–97.5)	16.20 (15.20–17.21)	16.16 (15.17–17.15)
New 4mo Rx	DALY averted, mean (SD)	16.21 (0.53)	16.20 (0.51)
	DALY averted, median (2.5–97.5)	16.22 (15.19–17.24)	16.18 (15.19–17.15)
	mean difference (%)	0.02 (0.06)	0.02 (0.06)
	ICER, median (2.5–97.5)	164 (CS-9,075)	129 (CS-8,824)
	ICER, calculated mean	1,472	1,220
GDP per capita		829	
Tanzania			
Current 6mo Rx	DALY averted, mean (SD)	13.66 (0.36)	12.97 (0.36)
	DALY averted, median (2.5–97.5)	13.66 (12.98–14.35)	12.96 (12.31–13.65)
New 4mo Rx	DALY averted, mean (SD)	13.68 (0.36)	13.00 (0.36)
	DALY averted, median (2.5–97.5)	13.67 (12.99–14.36)	12.99 (12.32–13.70)
	mean difference (%)	0.02 (0.04)	0.03 (0.05)
	ICER, median (2.5–97.5)	CS (CS-12,252)	39 (CS-3,937)
	ICER, calculated mean	CS	161
GDP per capita		695	

*Mo* months, *Rx* treatment, *DALY* disability-adjusted life years, *SD* standard deviation, *ICER* incremental cost effectiveness ration, *CS* cost saving, *GDP per capita* gross domestic product per capita

**Fig. 2:**
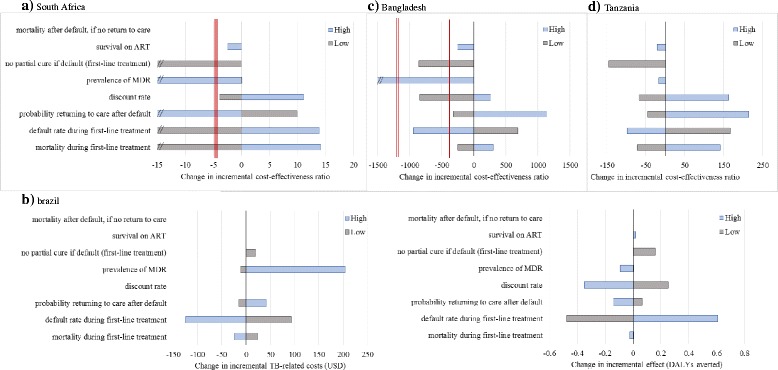
One-way sensitivity analysis by country. **a** South Africa (current scenario, drug price 1USD per day). We show variations in incremental cost-effectiveness ratio (ICER) to analyse the influence of different assumptions on our conclusions of cost-effectiveness. High/low refers to a higher/lower value of the parameter being considered compared to the baseline value. The x-axis shows the change in the ICER where 0 represents no change (ie baseline ICER). The double *red line* represents the change in ICER when the result is cost saving (i.e. negative ICERs). Negative ICERs are not at scale and this is indicated by a double slash. **b** Brazil (guidelines current, drug price 1USD per day). We show variations in incremental cost and incremental effect as opposed to changes in incremental cost-effectiveness ratio (ICER) because for Brazil, the ICER remains negative in this scenario (ie cost saving). The purpose is to investigate the impact of our assumptions on two components of the ICER: incremental cost and incremental effect. High/low refers to a higher/lower value of the parameter being considered compared to the baseline value. The x-axis shows the change in incremental costs or incremental effects (DALYs averted) compared to the baseline result (a negative value in incremental costs means less cost differentials, same applies to the DALYs), 0 represents no change (ie baseline). **c** Bangladesh (current scenario, drug price 1USD per day). We show variations in incremental cost-effectiveness ratio (ICER) to analyse the influence of different assumptions on our conclusions of cost-effectiveness. High/low refers to a higher/lower value of the parameter being considered compared to the baseline value. The x-axis shows the change in the ICER where 0 represents no change (ie baseline ICER). The single *red line* represents the change in ICER when the result becomes cost-effective (one GDP as willingness-to-pay threshold). The double *red line* represents the change in ICER when the result is cost saving (i.e. negative ICERs). Negative ICERs are not at scale and this is indicated by a double slash. **d** Tanzania (current scenario, drug price 1USD per day). We show variations in incremental cost-effectiveness ratio (ICER) to analyse the influence of different assumptions on our conclusions of cost-effectiveness. High/low refers to a higher/lower value of the parameter being considered compared to the baseline value. The x-axis shows the change in the ICER where 0 represents no change (ie baseline ICER)

Cost-effectiveness varies by setting when we assume the drugs for the shorter regimen to be priced at USD1 per day (Table 4). In South Africa, only the current scenario was not estimated to be cost saving but remained a cost-effective choice (mean USD13.6 per DALY averted). In Tanzania, new shortened regimens in the guideline scenario might be cost saving, but when ‘real world’ considerations are taken into account, extra investment is needed. However, this extra investment was cost-effective (mean USD161 per DALY averted). In contrast, in Bangladesh, across both scenarios, this investment was not cost-effective when compared to one GDP per capita as a WTP threshold (mean USD1,472 and 1,220 per DALY averted in guidelines and current scenarios, respectively), reflecting the trade-off between the higher drug prices assumed for the new regimen and low service delivery costs. In one-way sensitivity analyses, our conclusions were stable to most of the assumptions made (Fig. 2). Cost-effectiveness was most sensitive to existing health service costs for treatment delivery and default rates. In addition to these, in Brazil and South Africa (Fig. 2a and b), a higher prevalence of MDR resulted in an increase in cost savings, while a high probability of returning to care after default reduced the incremental benefits observed. In Bangladesh (Fig. 2c), a higher prevalence in MDR changed from an assessment of the new regimen not being cost-effective to being cost-saving. The new shortened regimen remained cost-effective in Tanzania under all analyses.

**Fig. 3:**
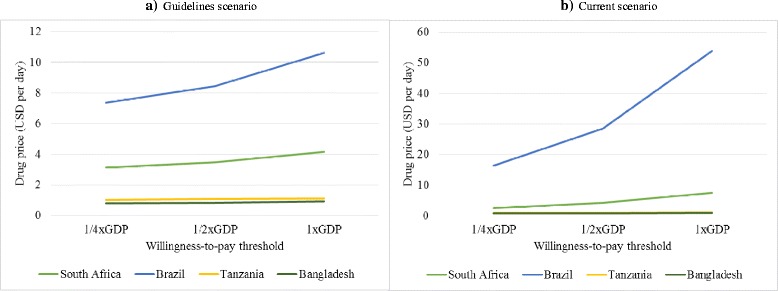
Estimated drug price per month at which the mean ICER (new regimen vs standard) crosses a particular CE threshold by country and scenario. **a** Guidelines scenario. **b** Current scenario. Drug price in the y-axis is the drug price at which the mean ICER crosses the WTP threshold. GDP: gross domestic product per capita

Using a threshold analysis (Fig. 3), we estimated that in settings with low treatment delivery costs (Bangladesh and Tanzania) the threshold price for achieving cost-effectiveness is approximately USD1 per day and it remains at this level if different assumptions of adherence to guidelines are made. In settings with higher treatment delivery costs (Brazil and South Africa), the threshold price varies substantially depending on how closely current practice adheres to guidelines. In general, the threshold drug price are reduced if treatment guidelines are totally adhered to (mainly due to a higher cost in health service delivery and better effect).

## Discussion

We present a four-country economic evaluation of the introduction of a new hypothetical four-month TB regimen compared to the current six-month regimen. The cost-effectiveness of the new shortened regimen for first-line TB treatment varies substantially by setting and current treatment practice. We find that a new non-inferior shortened regimen costing USD1 a day would likely be cost-saving in Brazil, South Africa, and Tanzania, but not in Bangladesh, assuming full adherence to treatment guidelines. Potential health service-related cost savings are modest and are lower when ‘real world’ treatment practice is taken into account. Patient incurred cost savings are substantial in all settings, especially when services are facility-based rather than community-based. Individual health benefits are positive but modest.

Our findings are line with other recent studies that, contrary to earlier efforts, suggest that the health benefits (including population level) of regimen shortening are likely to be limited [11]. However, in light of the post-2015 TB control strategy that aims for ‘no affected families facing catastrophic costs due to TB’ by 2025, we highlight the importance of the economic benefits of regimen shortening to patients. For example, in South Africa, 37% of TB patients were found to incur catastrophic costs; [17] and thus reducing costs by 25% may prevent considerable numbers falling into poverty because of contracting TB.

The difference in health service costs between settings highlights the importance of accounting for setting-specific resource use, health service and demand constraints. Previous efforts, have assumed that any health service costs could be substantial and if usefully channeled back into other TB services – may have substantial impact [9]. Our findings are more modest and variable, but it should be noted that any savings remain critical, in the current situation where most current TB control programmes are substantially underfunded [1]. Paradoxically, incorporating a ‘real world’ perspective results in new regimens having lower economic benefits. However, one of the reasons for poor guideline adherence may be substantial constraints to TB treatment delivery, and so even freeing up modest resources in these settings may be important to improve TB treatment delivery more generally.

Drug prices have an important impact on our findings. However, to date, there is no public information on what the future cost of such a regimen would be or whether it will vary by setting. Initial estimates of previously tested shortened regimens priced these regimens between USD10 and USD25 per month. We assumed a price of USD30 to provide a conservative point estimate of drug costs. However, as there is a high degree of uncertainty around price, we conducted a threshold analysis. Here we found that the threshold price per day was substantially higher in those settings where treatment delivery costs were higher (South Africa, USD10; Brazil, USD57); this threshold was sensitive to the adherence to guidelines assumptions. For those settings with lower treatment delivery costs (e.g. Tanzania and Bangladesh), USD1 per day was the maximum price for cost-effectiveness, but this threshold is sensitive to the uncertainty observed in effect estimates.

Our study has several limitations. We excluded benefits in children or in the prevention of downstream transmission and acquired resistance. The additional benefits due to transmission prevented has been estimated to be low in previous studies [11, 28], The benefits from reductions in acquired resistance are highly uncertain. This is in part due to lack of data on the probability of acquiring resistance, particularly where the precise regimen remains undefined. Once regimen profiles become available this will be an important consideration for further analyses. At this point, we are presenting conservative results, and it is possible that higher regimen prices may be cost-effectiveness once these benefits are taken into account. However, the additional benefits due to transmission prevented has been estimated to be low, while there is significant uncertainty on the effect of new regimens on future acquired resistance trends [11, 28]. With regards to benefits due to acquired resistance prevented, the magnitude of these benefits will depend exclusively on the drug regimen being introduced and will be an important consideration for further analyses. We did not include programme costs of new drug introduction (i.e. system-wide costs allowing the new regimen to be introduced, such as development of new treatment guidance or setting up new monitoring systems) nor did we examine the influence of alternative approaches to treatment observation going forward. In the case of the former, this exclusion may overestimate cost savings. However, any introduction cost, or startup cost, would be a one-off occurrence which would be discounted over time. In the case of the latter, where lower cost methods of observation are introduced in both base case and the alternative, cost savings may also be reduced.

## Conclusions

A four-month non-inferior first-line TB regimen is likely to be cost saving or cost-effective in many country settings. This benefit is more marked in middle income countries, like South Africa and Brazil, where health service delivery costs are higher. Adherence to TB treatment guidelines is a key determinant of cost-effectiveness when considering the introduction of shortened regimens. In low income countries, like Tanzania and Bangladesh, drug price is likely to be critical for cost-effectiveness. In terms of the post-2015 global TB targets, the most notable benefit of shortened regimens is to reduce the economic burden on households. In reaching these conclusions, we adopted an approach that considers individual and health service utilisation characteristics as well as societal costs using country-specific information, allowing us to tailor the analysis and conclusions to specific ‘real world’ settings.

## Additional files

Additional file 1: Supporting information (SI). Cost and cost-effectiveness of tuberculosis treatment shortening: a model-based analysis Gomez GB et al. August 2016. (DOCX 1955 kb)
Additional file 2: CHEERS checklist and technical appendix including additional results. (DOCX 16 kb)

## Abbreviations

ART: Antiretroviral treatment
CP: Continuation phase of a six-month first line regimen (four last months)
CS: Cost saving
DALY: Disability-adjusted life-year
DOT: Directly observed therapy
DST: Drug sensitivity testing
GDP: Gross domestic product
HIV: Human immunodeficiency virus
ICER: Incremental cost-effectiveness ratio
IP: Intensive phase of a six-month first line regimen (first two months)
MDR-TB: Multi-drug resistant tuberculosis
mo: Months
Pr: Probability
RIF: Rifampin
Rx: Treatment
SD: Standard deviation
TB: Tuberculosis
USD: US dollar
WTP: Willingness to pay
