# Supplementary material for: Cost and cost-effectiveness of tuberculosis treatment shortening: a model-based analysis
Source: BMC Infect Dis. 2016 Dec 1;16:726. doi: 10.1186/s12879-016-2064-3 (PMC5131398; doi:10.1186/s12879-016-2064-3)
Supplement: Additional file 1: — Supporting information (SI). Cost and cost-effectiveness of tuberculosis treatment shortening: a model-based analysis Gomez GB et al. August 2016. (DOCX 1955 kb) [file 12879_2016_2064_MOESM1_ESM.docx]

Supporting information (SI)

Cost and cost-effectiveness of tuberculosis treatment shortening: a model-based analysis

Gomez GB et al. August 2016

TABLE OF CONTENTS

[Technical information 2](#_Toc436395843)

[Setting 2](#_Toc436395844)

[Default 2](#_Toc436395845)

[Current Standard of Care 3](#_Toc436395846)

[Model Schematics 5](#_Toc436395847)

[Costs 6](#_Toc436395848)

[Disability adjusted life years 10](#_Toc436395849)

[Additional results 11](#_Toc436395850)

[References 25](#_Toc436395851)

# **Technical information**

## **Setting**

Bangladesh is a high TB and high MDR-TB burden country, with a low prevalence of HIV among TB patients (3%) and high mortality rates. With a population over 150 million people, access to laboratory analyses were reported to be 0.7 smear per 100,000 population and <0.1 culture or drug susceptibility testing per five million population in 2013 (1). Brazil is a high TB and high HIV burden country, with a prevalence of HIV among TB patients of 17%. With a population of approximately 200 million people, access to laboratory analyses were reported to be 1.5 smear per 100,000 population and 8.6 and one culture and drug susceptibility testing per five million population in 2013, respectively (1). South Africa is a high TB, high HIV, and high MDR-TB burden country, with a prevalence of HIV among TB patients of 62% and high mortality rates. With a population over 50 million people, access to laboratory analyses were reported to be 0.4 smear per 100,000 population and 1.4 culture and drug susceptibility testing per five million population in 2013 (1). Tanzania is a high TB and high HIV burden country, with a prevalence of HIV among TB patients of 37%. With a population of just under 50 million people, access to laboratory analyses were reported to be 1.9 smear per 100,000 population and 0.5 and 0.1 culture and drug susceptibility testing per five million population in 2013, respectively (1).

**Table S1. Estimated epidemiological burden of TB, 2014 (adapted from(1)).**

|  | Mortality  (rate per 100,000 excluding HIV) | Prevalence  (rate per 100,000 including HIV) | Incidence  (rate per 100,000 including HIV) | Incidence among HIV positive  (rate per 100,000) | % of TB cases with MDR-TB |
| --- | --- | --- | --- | --- | --- |
|  | *Best [low - high]* | *Best [low - high]* | *Best [low - high]* | *Best [low - high]* | *Best [low - high]* |
| Bangladesh | 51 [33–69] | 402 [210–656] | 224 [199–253] | 0.42 [0.23–0.67] | New: 1.4 [0.7–2.5]  Retreatment: 29 [24–34] |
| Brazil | 2.2 [1.3–3.4] | 57 [27–99] | 46 [41–52] | 6.5 [6.5–6.6] | New: 1.4 [1–1.8]  Retreatment: 7.5 [5.7–9.9] |
| South Africa | 48 [28–73] | 715 [396–1,126] | 860 [776–980] | 520 [464–594] | New: 1.8 [1.4–2.3]  Retreatment: 6.7 [5.4–8.2] |
| Tanzania | 12 [7–17] | 172 [92–277] | 164 [157–170] | 61 [58–63] | New: 1.1 [0.5–2]  Retreatment: 3.1 [0.9–7.9] |

HIV, human immunodeficiency virus; MDR, multi-drug resistant TB. “New” refers to MDR-TB prevalence among new patients; “Retreatment” refers to MDR-TB prevalence among retreatment patients.

## **Default**

The duration of treatment before default as well as the duration of the default period determines the treatment algorithm to be followed once the patient returns to care. A patient is considered a “default” after stopping treatment for two months. These considerations are laid out in table S2.

**Table S2 - Treatment guidelines for patients returning to care after stopping treatment.**

| **Treatment duration at time of stopping:** | **Stop treatment for:** | **Treatment recommendation when returning to care:** | **Tests recommended when returning to care:** |
| --- | --- | --- | --- |
| **Less than 5 months** | Less than 2 months | Continue treatment category I |  |
| **Less than 1 month** | 2 months or more | Restart treatment category I |  |
| **1 to 5 months** | 2 months or more | If smear positive, start treatment category II or follow Xpert results to determine further treatment | 3 sputa (or Xpert when available) |
| **5 to 6 months** | Any duration | If smear positive, start treatment category II or follow Xpert results to determine further treatment | 3 sputa (or Xpert when available) |

## **Current Standard of Care**

The current TB treatment guidelines from the four countries of interest are summarised in Table S3, including: DST algorithms, first-line therapy recommended for patients category I (new patient with smear positive pulmonary TB disease), category II (relapse, treatment failure, or smear positive patient after default), and III (new patient with smear negative pulmonary TB disease and/or extrapulmonary involvement); monitoring; standardised MDR treatment; DOT recommendations; and ART eligibility for HIV/TB patients.

**Table S3 - Current standard of care.**

|  | **South Africa** (2)(3)(4)(5) | **Tanzania** (6)(7) | **Bangladesh** (8)(9) | **Brazil** (10)(11) |
| --- | --- | --- | --- | --- |
| **Treatment: First-line therapy, category I and III** | Intensive: 2 months (FDC: RHZE) Continuation: 4 months (FDC: RH) | Intensive: 2 months (FDC: RHZE) Continuation: 4 months (FDC: RH); if DOT. If monthly supplied and self-administered, then continuation phase is 6 months (FDC: EH) | Intensive: 2 months (FDC: RHZE) Continuation: 4 months (FDC: RH) | Intensive: 2 months (FDC: RHZE) Continuation: 4 months (FDC: RH) |
| **Treatment: First-line therapy, category II†** | n/a | Intensive: 2 months (S (i.m.) + FDC: RHZE) + 1mo FDC: RHZE Continuation: 5 months (FDC: RH+E) | Intensive: 2 months (S (i.m.) + FDC: RHZE) + 1mo FDC: RHZE Continuation: 5 months (FDC: RH + E) | n/a |
| **Monitoring: category I and III** | 2 sputa before the end of second months. If positive, culture and 1 more month of intensive phase treatment and 2 sputa at the end. Start continuation phase (while waiting for culture results). Then at the end of month 5, 2 sputa and check results of culture | 2 sputa before the end of second months. If positive, culture and 1 more month of intensive phase treatment and 2 sputa at the end. Start continuation phase (while waiting for culture results). Then at the end of month 5, 2 sputa: if positive, culture and DST. Start category II regimen | 2 sputa before the end of second months. If positive, culture and 1 more month of intensive phase treatment and new sputa at the end. Start continuation phase, regardless of the smear result. If positive smear at the end of treatment, then failure is recorded and category II regimen started | 2 sputa specimen should be examined at the end of month 2, 4 and 6. If positive at month 2, culture should be done. |
| **Monitoring: category II** | n/a | 2 sputa before the end of third month. Culture should be done if positive results. 1 more month of intensive phase treatment. If still smear positive at month 4, start continuation phase (while waiting for culture results). At the end of month 7, sputum and check results of culture. | 2 sputa at the end of month 3, 5 and 8. If smear positive at month 3, should continue the intensive phase for one more month. 2 sputa after one month of extended intensive phase, start continuation phase. If smear positive at month 5, continue Category II treatment (while waiting for DST results). If smear positive at completion, referral. | n/a |
| **Standardised MDR treatment** | Intensive: at least 6 months (guided by TB culture conversion) of Kanamycin, ethionamide, pyrazinamide, levofloxacin, terizidone.  Continuation: at least 18 months of ethionamide, pyrazinamide, levofloxacin, terizidone.  Pyridoxine (VitB6) daily to patients on terizidone. | Intensive: at least 6 months of Kanamycin, ethionamide, pyrazinamide, levofloxacin, terizidone.  Continuation: at least 18 months of ethionamide, pyrazinamide, levofloxacin, terizidone.  Pyridoxine (VitB6) daily to patients on terizidone. | Intensive: at least 6 months of Kanamycin, ethionamide, pyrazinamide, levofloxacin, terizidone.  Continuation: at least 18 months of ethionamide, pyrazinamide, levofloxacin, terizidone.  Pyridoxine (VitB6) daily to patients on terizidone. | Intensive (6 months) of streptomycin, ethambutol, levofloxacin, pyrazinamide, terizidone.  Continuation (12 months) ethambutol, levofloxacin, terizidone.  Pyridoxine (VitB6) daily to patients on terizidone. Extend to 24 months if necessary. |
| **DOT recommendation** | Community, clinic, workplace, and friend/family DOT available. | DOT in clinic or at home under supervision of the supporter of choice. | First dose at health facility then referred. DOT provider: facility-/community-based health worker or trained community member. | Clinic- or home-based. |
| **ART for HIV/TB co-infected patients** | ART should be initiated for all people living with HIV with active TB disease irrespective of CD4 cell count. | ART should be initiated for all people living with HIV with active TB disease irrespective of CD4 cell count. | ART should be initiated for all people living with HIV with active TB disease irrespective of CD4 cell count. | ART should be initiated for people living with HIV with active TB disease at CD4 cell count of 500 cells/mL. |

†category II: category II treatment and the use of streptomycin may be phased out in the future. However, when shown, current guidelines still recommend this regimen.

R, rifampicin; H, isoniazid; Z, pyrazinamide; E, ethambutol; DOT, directly observed therapy; DST, drug susceptibility testing; S, streptomycin; i.m., intramuscular; Vit, vitamin; FDC, fixed dose combination; ART, antiretroviral therapy; HIV, human immunodeficiency virus.

## **Model Schematics**

**Figure S1 - Outcomes during treatment, model diagramme**.


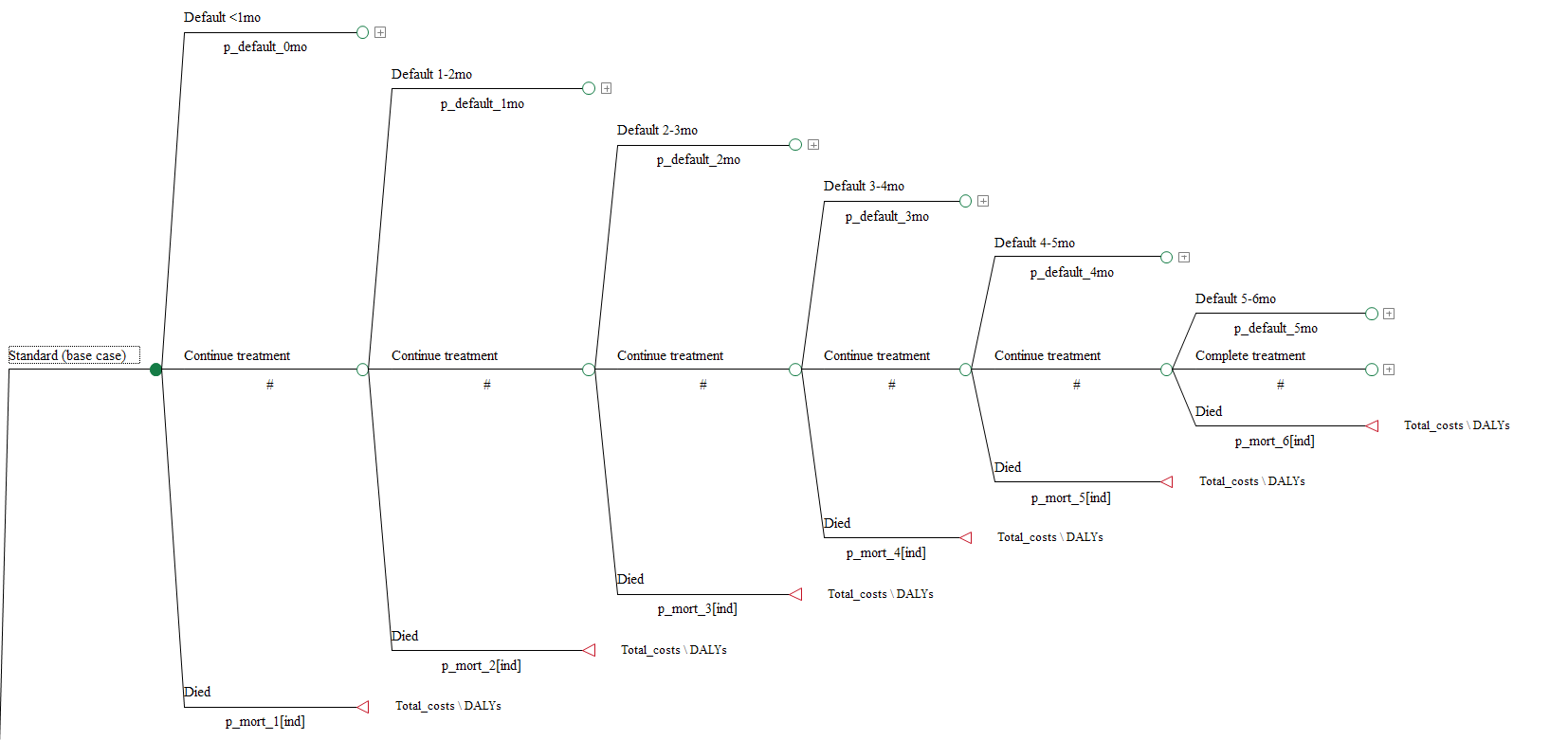


**Figure S2 - Outcomes after default, model diagramme.**


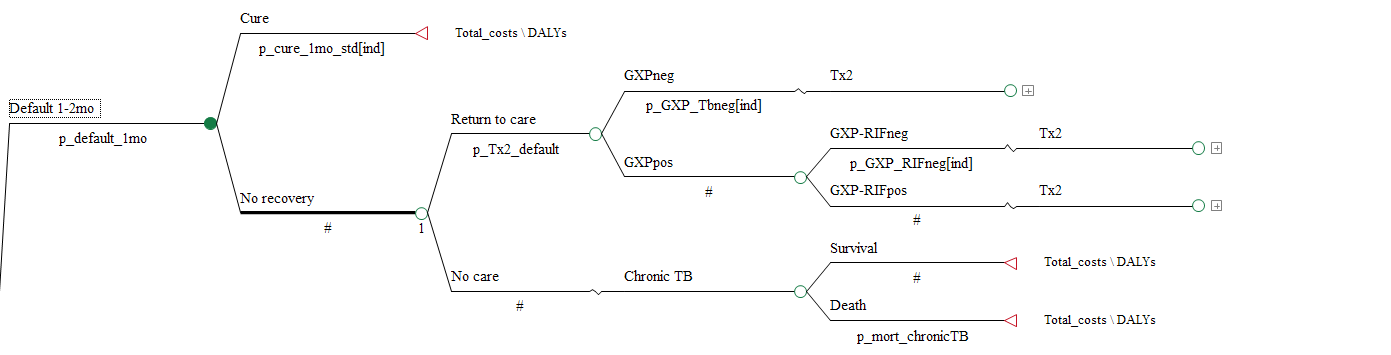


**Figure S3 - Outcomes after treatment completion, model diagramme.**


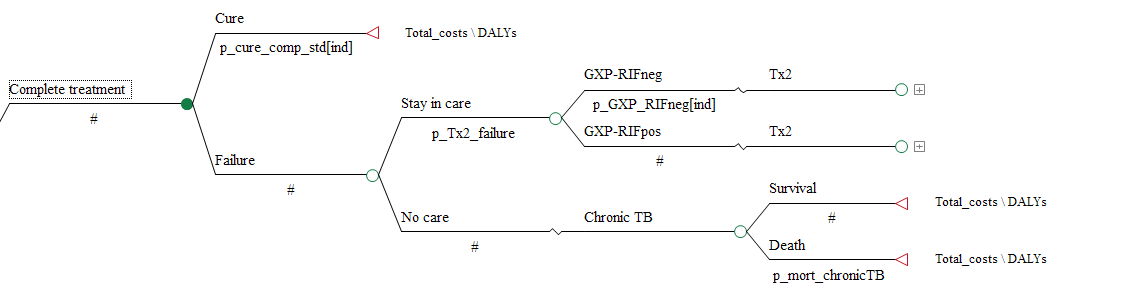


## **Costs**

**Table S4. Summary of data collection methods: costs**

|  | Health service costs | Patient costs |
| --- | --- | --- |
| South Africa | Estimating health service costs was part of the “Xpert for TB: Evaluating a New Diagnostic” (XTEND) trial design in South Africa (12). For the purposes of estimating the cost of drug sensitive TB treatment, eight out of ten XTEND study clinics were purposively selected based on the number of annual primary health care visits and proximity to patient’s homes. In addition, we aimed to get a representative sample of rural and urban facilities. Both recurrent (personnel, overheads, and maintenance) and capital costs (buildings, equipment & furniture, and training) were assessed using an ingredients approach – using a primarily bottom-up method. The only exception was non-personnel recurrent expenditure in clinics (i.e. overheads), for which only aggregated expenditure data were available, and a top-down allocation method used. Two methodologies used to estimate staff time use. Weekly time sheets, interviews and clinic cost data included the Provincial Administration were used to collect the data. | Estimating patient-related costs in a longitudinal cohort was part of the “Xpert for TB: Evaluating a New Diagnostic” (XTEND) trial design in South Africa. Full methods of data collection and analysis can be found elsewhere (13). Briefly, ten public health clinics across four provinces were purposefully selected from the trial sites to generate a representative sample of rural and urban sites, facilities with different workloads, plus the location of the clinics relative to the communities served. Respondents were asked about the costs incurred during their last visit; per period costs were then calculated by multiplying the cost per visit against the number of visits reported during that period. Individual income was estimated by asking detailed questions about locally-relevant income categories. Data about income from government grants and charity donations was also explicitly collected. In total, 351 TB suspects and 175 TB patients were included in the analysis. |
| Tanzania | We collected primary data on health service costs in Tanzania. Our costing activities were part of the study: Improving the health systems response to chronic diseases in Africa. We purposely selected six facilities providing care to TB patients in the Mwanza region. Costs were estimated using an ingredient approach. We included capital costs (building, equipment, furniture) and recurrent costs (labour, supplies, utilities, drugs, and monitoring and evaluation). In each participating clinic, we interviewed personnel and observed consultations to estimate the resource use for each input; we also reviewed financial and activity reporting. We also sourced costs of drugs for ART from this study to inform our ART cost estimates. | We sourced patient-related costs from a cross-sectional study on TB patient costs and perceptions, part of the TB Alliance grant portfolio. Full methods of data collection and analysis can be found elsewhere (14). Briefly, in Tanzania, the study was conducted at 5 urban and 12 rural health facilities in the Mwanza region. Remote and less remote areas that were representative of the national poverty and rural/urban profiles were selected from TB registers. In each area, male and female patients were randomly selected from TB registers until a set proportion of male and female patients aged >18 years was attained. Patients were interviewed at 16 weeks or later in their treatment, but no later than 2 months after treatment completion. Patients were asked to recall costs incurred during the first 2 months of treatment (intensive phase) separately from costs incurred during the 2 months leading up to the date of interview (continuation phase). Guardian/care givers costs were included as were also travel and other costs, such as charges for tests, administrative charges, costs associated with hospital admissions and costs of other medicines and supplements. Lost productivity was calculated as income lost due to TB for the patients and their guardians who would otherwise be in paid employment (i.e., excluding students, pensioners and those who were unemployed before the episode of TB). Any reimbursements received by patients through insurance would have been deducted, but none were recorded. The final sample was 94 patients. |
| Bangladesh | We collected primary data on health service costs in Bangladesh. Full details of our methods are presented elsewhere (15). Briefly, in an effort to represent the diversity of service delivery across Bangladesh, eight clinics were selected by overall patient volume (high /low) as well as reflecting a mix of rural, peri-rural and urban settings in four divisions across Bangladesh: Dhaka region, Chittagong, Rajshahi and Rangpur. We collected primary data on the delivery of first-line TB treatment using a combination of bottom-up and top-down methods. Details on overhead costs and key prices (such as salaries) were gathered and allocated using financial records and registers from local clinics and the national TB program offices in Dhaka. Observations of practice allowed allocation of time and other resources to activities related to TB diagnosis, monitoring, treatment, etc. We collected data on staff time use, relying on a mixture of observation and staff interview. Time use was then combined with salary costs to estimate the personnel or human resource cost associated with each procedure. Time use data was also used to assign overhead costs. | We sourced patient-related costs from a cross-sectional study on TB patient costs and perceptions, part of the TB Alliance grant portfolio. Full methods of data collection and analysis can be found elsewhere (14). In Bangladesh, the study was conducted in two upazilas (subdistricts) of each of the six districts, which were representative of the national situation in terms of population density, poverty and rural/urban profiles. The sampling and data collection methods were the same as for Tanzania. The final sample was 96 patients. |
| Brazil | We collected primary data on health service costs in Brazil. Full detailed methods are available elsewhere (16). Briefly, we purposively selected four geographical areas to obtain balance between the municipality of Rio and peri-urban areas. We then randomly sampled 12 facilities from a census of all eligible facilities currently treating at least 50 TB patients (excluding hospitals), stratified by workload (ratio of TB patients per healthcare workers) and type of clinic (family health clinics, health post, and mixed facilities). Costs from a healthcare provider’s perspective were estimated using an ingredient approach. We included capital costs (building, equipment, furniture) and recurrent costs (labour, supplies, utilities, drugs, and monitoring and evaluation). In each participating clinic, we interviewed personnel and observed consultations to estimate the resource use for each input; we also reviewed financial and activity reporting. | We collected primary data on patient costs in Brazil. Full detailed methods are available elsewhere (16). Briefly, we purposively selected four geographical areas to obtain balance between the municipality of Rio and peri-urban areas. We then randomly sampled 12 facilities from a census of all eligible facilities currently treating at least 50 TB patients (excluding hospitals), stratified by workload (ratio of TB patients per healthcare workers) and type of clinic (family health clinics, health post, and mixed facilities). All TB patients attending (or having home visits by a community health worker) at a participating clinic on their 5th or 6th month of treatment were invited to participate. After obtaining informed consent, data on demographics, assets, type of TB treatment, out-of-pocket expenditures, productive hours lost, coping costs (selling of assets and borrowed money), and care giver’s costs were gathered. Indirect costs included productivity loss, coping and care giver’s costs; direct costs included all out-of-pocket expenditure. With regards to productivity loss associated with seeking care, we considered both time seeking care and days off-work to account for both the costs of illness and the costs of seeking treatment. A total of 126 patients were interviewed. |

**Table S5. Detailed costs for guidelines/current scenarios**

1. Guidelines

|  | **South Africa** | **Tanzania** | **Bangladesh** | **Brazil** |  |
| --- | --- | --- | --- | --- | --- |
| Healthcare provider costs | | | | |  |
| First-line treatment, IP, 1mo (excl drugs) | 200 (152-230.8) | 65.3 (24.1-106.6) | 16.6 (11.9-21.3) | 332.8 (116.8-479) | (12,16,15) |
| First-line treatment, CP, 1mo (excl drugs) | 53.9 (41-62.2) | 15.5 (7.1-24) | 11.1 (7.6-14.5) | 332.8 (116.8-479) | (12,16,15) |
| Drugs, first-line, IP, 1mo | 16.3 | 6.3 | 6.6 (5.6-7.6) | 7.4 (6.4-8.5) | (12,16,15,17) |
| Drugs, first-line, CP, 1mo | 18.9 | 2.1 | 3 (2.8-3.3) | 4.2 (2.8-5.5) | (12,16,15,17) |
| Presumptive treatment of bacterial infection | 17.6 (12.9-20.6) | 6.5 (3.3-9.1) | 2.1 (0.9-3.3) | 5.7 (2.5-8.4) | (12,16,15) |
| Retreatment: all | n/a | 429.2 (309.8-548.7) | 212.7 (159.6-265.9) | n/a | (12,16,15) |
| MDR treatment: all | 10214.7 (8618.6-24579.6) | 2507.2 (2453.8-2560.6) | 4261.8 (3835.6-4688) | 5222.5 (4799.5-5348) | (12,16,15) |
| ART cost in year 1 | 1127.6 (1116.6-1138.5) | 314.9 (283.4-346.4) | 800 (720-880) | 5875 (5287.5-6462.5) | (18–23) |
| ART cost per year (after year 1) | 639.1 (575.2-703) | 276.7 (249.1-304.4) | 600 (540-660) | 5875 (5287.5-6462.5) | (18–23) |
| Xpert | 21.6 (14.6-28.4) | 22.9 (20.6-25.2) | 16.4 (14.8-18.1) | 20.6 (18.6-22.7) | (12,16,15) |
| Smear test | 7.9 (5.1-10.6) | 1.9 (1.7-2.1) | 2.7 (1.7-3.7) | 1.7 (1.6-1.9) | (12,16,15) |
| culture and DST | 53.9 (38-69.8) | 27.6 (24.9-30.4) | 22.4 (20.2-24.6) | 24.9 (22.4-27.4) | (12,16,15) |
| x-ray | 24.1 (21.7-26.5) | 4 (3-5) | 4 (3-5) | 2.5 (0-5) | (12,16,15) |
| Patient costs | | | | |  |
| First-line treatment, IP, 1mo | 148.7 (86.7-163.5) | 185.6 (167.1-204.2) | 314.4 (283-345.9) | 39.6 (8.3-130.9) | (13,14,16) |
| First-line treatment, CP, 1mo | 116.8 (33.6-128.5) | 44 (39.6-48.4) | 30.7 (27.6-33.8) | 39.6 (8.3-130.9) | (13,14,16) |
| Presumptive treatment of bacterial infection | 23.7 (21.3-26) | 22.3 (20.1-24.6) | 7.8 (7.1-8.6) | 19.2 (3.8-42) | (13,14,16) |
| Retreatment: all | n/a | 354.1 (318.7-389.5) | 134.6 (121.1-148) | n/a | (13,14,16) |
| MDR treatment: all | 3318.5 (2986.6-3650.3) | 454 (408.6-499.4) | 213 (191.7-234.3) | 279.6 (101.7-1141.7) | (13,14,16) |
| ART cost in year 1 | 106.2 (95.6-116.8) | 24 (21.6-26.4) | 7.6 (6.9-8.4) | 23.2 (3.9-43) | (24) |
| ART cost per year (after year 1) | 84.9 (76.5-93.4) | 9.6 (8.7-10.6) | 3.1 (2.8-3.4) | 9.3 (1.6-17.2) | (24) |
| Cost per visit to healthcare facility | 7.8 (7-8.6) | 4.8 (4.4-5.3) | 1.6 (1.4-1.7) | 4.7 (0.8-8.6) | (13,14,16) |

1. Current

|  | **South Africa** | **Tanzania** | **Bangladesh** | **Brazil** | **Reference** |
| --- | --- | --- | --- | --- | --- |
| Healthcare provider costs | | | | |  |
| First-line treatment, IP, 1mo (excl drugs) | 60.5 (39.4-95.8) | 34.8 (24.1-45.4) | 16.6 (11.9-21.3) | 132.8 (59.1-285.1) | (12,16,15) |
| First-line treatment, CP, 1mo (excl drugs) | 16.3 (10.7-25.8) | 15.6 (7.1-24) | 11.1 (7.6-14.5) | 132.8 (59.1-285.1) | (12,16,15) |
| Drugs, first-line, IP, 1mo | 16.30 | 6.30 | 6.6 (5.6-7.6) | 7.4 (6.4-8.5) | (12,16,15,17) |
| Drugs, first-line, IP, 1mo | 18.9 | 2.1 | 3 (2.8-3.3) | 4.2 (2.8-5.5) | (12,16,15,17) |
| Presumptive treatment of bacterial infection | 17.6 (12.9-20.6) | 6.5 (3.3-9.1) | 2.1 (0.9-3.3) | 5.7 (2.5-8.4) | (12,16,15) |
| Retreatment: all | n/a | 429.2 (309.8-548.7) | 212.7 (159.6-265.9) | n/a | (12,16,15) |
| MDR treatment: all | 10214.7 (8618.6-24579.6) | 2507.2 (2453.8-2560.6) | 4261.8 (3835.6-4688) | 5222.5 (4799.5-5348) | (12,16,15) |
| ART cost in year 1 | 1127.6 (1116.6-1138.5) | 314.9 (283.4-346.4) | 800 (720-880) | 5875 (5287.5-6462.5) | (18–23) |
| ART cost per year (after year 1) | 639.1 (575.2-703) | 276.7 (249.1-304.4) | 600 (540-660) | 5875 (5287.5-6462.5) | (18–23) |
| Xpert | 21.6 (14.6-28.4) | 22.9 (20.6-25.2) | 16.4 (14.8-18.1) | 20.6 (18.6-22.7) | (12,16,15) |
| Smear test | 7.9 (5.1-10.6) | 1.9 (1.7-2.1) | 2.7 (1.7-3.7) | 1.7 (1.6-1.9) | (12,16,15) |
| culture and DST | 53.9 (38-69.8) | 27.6 (24.9-30.4) | 22.4 (20.2-24.6) | 24.9 (22.4-27.4) | (12,16,15) |
| x-ray | 24.1 (21.7-26.5) | 4 (3-5) | 4 (3-5) | 2.5 (0-5) | (12,16,15) |
| Patient costs | | | | |  |
| First-line treatment, IP, 1mo | 60 (35-66) | 143.9 (138.7-149.1) | 314.4 (283-345.9) | 39.6 (8.3-130.9) | (13,14,16) |
| First-line treatment, CP, 1mo | 27 (8-30) | 40.5 (37-44) | 30.7 (27.6-33.8) | 39.6 (8.3-130.9) | (13,14,16) |
| Presumptive treatment of bacterial infection | 23.7 (21.3-26) | 22.3 (20.1-24.6) | 7.8 (7.1-8.6) | 19.2 (3.8-42) | (13,14,16) |
| Retreatment: all | n/a | 354.1 (318.7-389.5) | 134.6 (121.1-148) | n/a | (13,14,16) |
| MDR treatment: all | 3318.5 (2986.6-3650.3) | 454 (408.6-499.4) | 213 (191.7-234.3) | 279.6 (101.7-1141.7) | (13,14,16) |
| ART cost in year 1 | 106.2 (95.6-116.8) | 24 (21.6-26.4) | 7.6 (6.9-8.4) | 23.2 (3.9-43) | (24) |
| ART cost per year (after year 1) | 84.9 (76.5-93.4) | 9.6 (8.7-10.6) | 3.1 (2.8-3.4) | 9.3 (1.6-17.2) | (24) |
| Cost per visit to healthcare facility | 7.8 (7-8.6) | 4.8 (4.4-5.3) | 1.6 (1.4-1.7) | 4.7 (0.8-8.6) | (13,14,16) |

IP, intensive phase; CP, continuation phase; mo, month; excl, excluding; ART, antiretroviral treatment; MDR, multidrug resistant; DST, drug resistance testing.

## **Disability adjusted life years**

**Table S6 – DALY assumptions.**

| **Variable** | | **Value** | **Reference** |
| --- | --- | --- | --- |
| **Average age of onset of TB (years)** | South Africa | 38.1 | (25) |
|  | Brazil | 36.5 | (26) |
|  | Bangladesh | 46.26 | (27) |
|  | Tanzania | 47.18 | (6) |
| **Survival without cure for TB (years)** | HIV negative, smear negative | 7.36 | * |
|  | HIV negative, smear positive | 2.74 | * |
|  | HIV positive, smear negative | 0.83 | * |
|  | HIV positive, smear positive | 0.5 | * |
| **Survival (years)** | HIV/AIDS, no ART | 3 | (28–30) |
|  | HIV/AIDS, on ART | 12.9 | (28–30) |
| **Disability weights** | TB but no HIV | 0.331 | (31) |
|  | TB and HIV | 0.399 | (31) |
|  | HIV pre AIDS, symptomatic | 0.221 | (31) |
|  | HIV/AIDS, on ART | 0.053 | (31) |
|  | AIDS, no ART | 0.547 | (31) |

*Calculated from mortality assumptions in the model.

# **Additional results**

**Table S7. Detailed costs – mean cost per TB patient by country, payer, and scenario (excluding and including ART-related costs).**

| **Payer*** | **Regimen** |  | **Guidelines** | **Current** |
| --- | --- | --- | --- | --- |
| *South Africa* | | | | |
| **Health services, TB** | 6mo regimen | mean (SD) | 1165.5 (163) | 563 (84.2) |
|  |  | median (2.5-97.5) | 1137.3 (912.8-1528.1) | 552.9 (430-741.4) |
|  | 4mo regimen | mean (SD) | 1145.4 (161.6) | 610.4 (84) |
|  |  | median (2.5-97.5) | 1120.1 (897.8-1500.3) | 597.1 (478.9-787.6) |
|  |  | **difference in means (%)** | -20.1 (-1.7) | 47.5 (8.4) |
| **Patient, TB** | 6mo regimen | mean (SD) | 746.9 (84.2) | 268.4 (22) |
|  |  | median (2.5-97.5) | 756.6 (555.7-879.3) | 270 (223.3-307.1) |
|  | 4mo regimen | mean (SD) | 564.8 (47) | 222.4 (15.6) |
|  |  | median (2.5-97.5) | 566.8 (468.4-654.9) | 223 (190.8-250.3) |
|  |  | **difference in means (%)** | -182.1 (-24.4) | -46 (-17.1) |
| **Health services, incl. ART** | 6mo regimen | mean (SD) | 3093.7 (151.2) | 1995.2 (80) |
|  |  | median (2.5-97.5) | 3090 (2812.8-3390.7) | 1992.9 (1848.2-2148.3) |
|  | 4mo regimen | mean (SD) | 3096.6 (148.7) | 2002.4 (79.6) |
|  |  | median (2.5-97.5) | 3092.1 (2818.4-3392.4) | 1999.9 (1853.7-2152.5) |
|  |  | **difference in means (%)** | 2.9 (0.1) | 7.2 (0.4) |
| **Patient, incl. ART** | 6mo regimen | mean (SD) | 396.8 (19.4) | 255.2 (10.4) |
|  |  | median (2.5-97.5) | 395.6 (361.1-437.5) | 255.6 (235.2-274.9) |
|  | 4mo regimen | mean (SD) | 397.1 (19.2) | 256.1 (10.4) |
|  |  | median (2.5-97.5) | 396.3 (361.7-437.1) | 256.4 (236.1-275.7) |
|  |  | **difference in means (%)** | 0.4 (0.1) | 0.9 (0.4) |
| **Total societal** | 6mo regimen | mean | 1912.4 (193.2) | 148.1 (1789.5) |
|  | 4mo regimen | mean | 1710.3 (179.6) | 132.5 (1582.9) |
|  |  | **difference in means (%)** | -202.2 (-10.6) | 1.5 (0.2) |
| **Total societal, incl. ART** | 6mo regimen | mean | 5402.9 | 3081.7 |
|  | 4mo regimen | mean | 5204.0 | 3091.4 |
|  |  | **difference in means** | -198.9 | 9.7 |
| *Brazil* | | | | |
| **Health services, TB** | 6mo regimen | mean (SD) | 1972.5 (319.7) | 950.2 (176.6) |
|  |  | median (2.5-97.5) | 1975.2 (1333.6-2566.4) | 946.8 (641.9-1314.6) |
|  | 4mo regimen | mean (SD) | 1509.1 (201.8) | 790.3 (121.2) |
|  |  | median (2.5-97.5) | 1512.5 (1117-1870) | 785.8 (576.9-1042.5) |
|  |  | **difference in means (%)** | -463.3 (-23.5) | -159.9 (-16.8) |
| **Patient, TB** | 6mo regimen | mean (SD) | 371 (110.9) | 338.7 (100.1) |
|  |  | median (2.5-97.5) | 359.7 (184.6-607.9) | 331.8 (167.7-544.6) |
|  | 4mo regimen | mean (SD) | 254.9 (62) | 236.4 (59.6) |
|  |  | median (2.5-97.5) | 251.1 (142.7-387.5) | 233.1 (133.9-360.3) |
|  |  | **difference in means (%)** | -116.1 (-31.3) | -102.3 (-30.2) |
| **Health services, incl. ART** | 6mo regimen | mean (SD) | 6959.3 (320) | 3539.1 (201.9) |
|  |  | median (2.5-97.5) | 6961.4 (6320.4-7569.1) | 3528 (3178.6-3970.8) |
|  | 4mo regimen | mean (SD) | 6963.8 (319.9) | 3572.7 (201.4) |
|  |  | median (2.5-97.5) | 6966.1 (6330.3-7563.8) | 3569.9 (3211.7-3998.6) |
|  |  | **difference in means (%)** | 4.5 (0.1) | 33.6 (1) |
| **Patient, incl. ART** | 6mo regimen | mean (SD) | 12.2 (3.7) | 6.4 (1.9) |
|  |  | median (2.5-97.5) | 12.1 (5.5-19.5) | 6.4 (2.9-9.8) |
|  | 4mo regimen | mean (SD) | 12.3 (3.7) | 6.4 (1.9) |
|  |  | median (2.5-97.5) | 12.2 (5.5-19.6) | 6.4 (2.9-9.9) |
|  |  | **difference in means (%)** | 0 (0.1) | 0.1 (0.9) |
| **Total societal** | 6mo regimen | mean | 2343.5 (340.3) | 320.7 (2303.2) |
|  | 4mo regimen | mean | 1764.1 (214.1) | 224.2 (1711) |
|  |  | **difference in means (%)** | -579.4 (-24.7) | -262.2 (-20.3) |
| **Total societal, incl, ART** | 6mo regimen | mean | 9315.1 | 4834.3 |
|  | 4mo regimen | mean | 8740.2 | 4605.8 |
|  |  | **difference in means** | -574.9 | -228.5 |
| *Bangladesh* | | | | |
| **Health services, TB** | 6mo regimen | mean (SD) | 125.7 (8.5) | 109.2 (7.3) |
|  |  | median (2.5-97.5) | 125.6 (110-143.7) | 109.1 (95.2-123.9) |
|  | 4mo regimen | mean (SD) | 200.6 (6.5) | 184.4 (5.3) |
|  |  | median (2.5-97.5) | 200.3 (188.9-214.2) | 184.4 (174.2-194.9) |
|  |  | **difference in means (%)** | 75 (59.7) | 75.2 (68.9) |
| **Patient, TB** | 6mo regimen | mean (SD) | 747.1 (26) | 747.3 (25.6) |
|  |  | median (2.5-97.5) | 747.4 (698.4-796.4) | 746.5 (700.3-795.3) |
|  | 4mo regimen | mean (SD) | 703.3 (25.8) | 701.9 (26.4) |
|  |  | median (2.5-97.5) | 703.6 (653.4-750.7) | 702.3 (650.4-750) |
|  |  | **difference in means (%)** | -43.8 (-5.9) | -45.4 (-6.1) |
| **Health services, incl. ART** | 6mo regimen | mean (SD) | 101.9 (8.2) | 101.8 (8.3) |
|  |  | median (2.5-97.5) | 101.7 (86.6-118.2) | 101.4 (85.6-118.2) |
|  | 4mo regimen | mean (SD) | 102 (8.3) | 102 (8.5) |
|  |  | median (2.5-97.5) | 101.7 (86.2-118.6) | 101.7 (85.7-118.2) |
|  |  | **difference in means (%)** | 0 (0) | 0.2 (0.2) |
| **Patient, incl. ART** | 6mo regimen | mean (SD) | 0.6 (0) | 0.6 (0) |
|  |  | median (2.5-97.5) | 0.6 (0.5-0.6) | 0.6 (0.5-0.7) |
|  | 4mo regimen | mean (SD) | 0.6 (0) | 0.6 (0) |
|  |  | median (2.5-97.5) | 0.6 (0.5-0.6) | 0.6 (0.5-0.6) |
|  |  | **difference in means (%)** | 0 (0) | 0 (0.2) |
| **Total societal** | 6mo regimen | mean | 872.8 (27.2) | 27.2 (869.5) |
|  | 4mo regimen | mean | 904 (27) | 27 (902.1) |
|  |  | **difference in means (%)** | 31.2 (3.6) | 29.8 (3.5) |
| **Total societal, incl. ART** | 6mo regimen | mean | 975.3 | 958.9 |
|  | 4mo regimen | mean | 1006.5 | 988.8 |
|  |  | **difference in means** | 31.3 | 30.0 |
| *Tanzania* | | | | |
| **Health services, TB** | 6mo regimen | mean (SD) | 222.5 (36.7) | 152.8 (15.3) |
|  |  | median (2.5-97.5) | 222.9 (156.2-293.2) | 152.7 (122.5-183.9) |
|  | 4mo regimen | mean (SD) | 294.7 (34) | 223.8 (11) |
|  |  | median (2.5-97.5) | 295.5 (227.6-359.5) | 224.1 (200.9-245.6) |
|  |  | **difference in means (%)** | 72.3 (32.5) | 70.9 (46.4) |
| **Patient, TB** | 6mo regimen | mean (SD) | 548.4 (16.7) | 445.3 (6.9) |
|  |  | median (2.5-97.5) | 548.3 (517.5-579.7) | 445.3 (432-458.3) |
|  | 4mo regimen | mean (SD) | 475.2 (16.3) | 378.7 (5.4) |
|  |  | median (2.5-97.5) | 474.4 (446.5-504.6) | 378.9 (368.2-389) |
|  |  | **difference in means (%)** | -73.2 (-13.3) | -66.6 (-15) |
| **Health services, incl. ART** | 6mo regimen | mean (SD) | 651.7 (27.4) | 473.1 (20.5) |
|  |  | median (2.5-97.5) | 650.9 (598.7-705.3) | 472.8 (436.6-513.2) |
|  | 4mo regimen | mean (SD) | 652.1 (27.3) | 473.8 (20.6) |
|  |  | median (2.5-97.5) | 651.7 (600.5-705.2) | 473.9 (437.4-513.6) |
|  |  | **difference in means (%)** | 0.4 (0.1) | 0.7 (0.2) |
| **Patient, incl. ART** | 6mo regimen | mean (SD) | 25 (1) | 18.1 (0.8) |
|  |  | median (2.5-97.5) | 25 (23.2-27) | 18.1 (16.7-19.5) |
|  | 4mo regimen | mean (SD) | 25 (1) | 18.1 (0.8) |
|  |  | median (2.5-97.5) | 25 (23.3-27.1) | 18.2 (16.7-19.6) |
|  |  | **difference in means (%)** | 0 (0.1) | 0 (0.2) |
| **Total societal** | 6mo regimen | mean | 770.9 (41) | 38.8 (761.8) |
|  | 4mo regimen | mean | 770 (38.3) | 37.5 (763.3) |
|  |  | **difference in means (%)** | -0.9 (-0.1) | 4.3 (0.7) |
| **Total societal, incl. ART** | 6mo regimen | mean | 1447.6 | 1089.4 |
|  | 4mo regimen | mean | 1447.1 | 1094.5 |
|  |  | **difference in means** | -0.5 | 5.0 |

Incl, including; ART, antiretroviral treatment; mo, month; SD, standard deviation.

*Health services, TB costs refer to costs borne by the health service related only to TB care; Patient, TB costs refer to costs borne by the patients related only to TB disease; Health services, incl. ART costs refer to costs borne by the health service related to both TB and ART care; Patient, incl. ART costs refer to costs borne by the patients related to both TB and ART care; Total societal costs include costs related to TB care only borne by both the health service and patients; Total societal, incl. ART costs include costs related to both TB and ART care borne by both the health service and patients.

**Table S8. Benefits in a 10,000 individual cohort, low and high default rates by country**

1. South Africa

|  | | **Guidelines** | |  | **Current** | |
| --- | --- | --- | --- | --- | --- | --- |
|  |  | 6mo regimen | 4mo regimen |  | 6mo regimen | 4mo regimen |
| Initially detected as MDR patients | | 349 | 349 | 8% default | 374 | 374 |
|  | |  |  | 30% default | 320 | 320 |
| Started on first-line treatment | | 9,651 | 9,651 | 8% default | 9,626 | 9,626 |
|  | |  |  | 30% default | 9,680 | 9,680 |
|  | Complete | 9,011 | 9,042 | 8% default | 7,978 | 8,196 |
|  | |  |  | 30% default | 6,269 | 6,977 |
|  | Default | 155 | 124 | 8% default | 708 | 490 |
|  | |  |  | 30% default | 2,473 | 1,765 |
|  | Death | 485 | 485 | 8% default | 940 | 940 |
|  | |  |  | 30% default | 938 | 938 |
| Completed and cured | | 8,745 | 8,780 | 8% default | 7,726 | 7,927 |
|  | |  |  | 30% default | 6,073 | 6,752 |
| Defaulted but cured | | 90 | 66 | 8% default | 417 | 247 |
|  | |  |  | 30% default | 1,386 | 833 |
| Total MDR treatment initiated | | 361 | 362 | 8% default | 232 | 227 |
|  | |  |  | 30% default | 201 | 200 |
| Total survival events (chronic TB) | | 46 | 61 | 8% default | 140 | 130 |
|  | |  |  | 30% default | 252 | 235 |
| Death if of chronic TB cases | | 161 | 161 | 8% default | 372 | 351 |
|  | |  |  | 30% default | 728 | 658 |
| Death during MDR treatment | | 32 | 32 | 8% default | 15 | 15 |
|  | |  |  | 30% default | 21 | 21 |
| Total deaths | | 689 | 683 | 8% default | 1,350 | 1,329 |
|  | |  |  | 30% default | 1,744 | 1,675 |

| Overall default | 1.6% | 1.3% | 8% default | 7.4% | 5.1% |
| --- | --- | --- | --- | --- | --- |
|  |  |  | 30% default | 25.5% | 18.2% |
| Relative reduction, default | ref | 20.0% | 8% default | ref | 30.8% |
|  |  |  | 30% default | ref | 28.6% |
| Absolute reduction, default | ref | 0.3% | 8% default | ref | 2.3% |
|  |  |  | 30% default | ref | 7.3% |
| Mortality during first-line treatment | 5.0% | 5.0% | 8% default | 9.8% | 9.8% |
|  |  |  | 30% default | 9.7% | 9.7% |
| Total mortality | 6.9% | 6.8% | 8% default | 13.5% | 13.3% |
|  |  |  | 30% default | 17.4% | 16.8% |
| Relative reduction, mortality | ref | 0.9% | 8% default | ref | 1.6% |
|  |  |  | 30% default | ref | 4.0% |
| Absolute reduction, mortality | ref | 0.1% | 8% default | ref | 0.2% |
|  |  |  | 30% default | ref | 0.7% |

1. Tanzania

|  | | **Guidelines** | |  | **Current** | |
| --- | --- | --- | --- | --- | --- | --- |
|  |  | 6mo regimen | 4mo regimen |  | 6mo regimen | 4mo regimen |
| Initially detected as MDR patients | | 0 | 0 | 3% default | 0 | 0 |
|  | |  |  | 15% default | 0 | 0 |
| Started on first-line treatment | | 10,000 | 10,000 | 3% default | 10,000 | 10,000 |
|  | |  |  | 15% default | 10,000 | 10,000 |
|  | Complete | 9,478 | 9,531 | 3% default | 9,128 | 9,220 |
|  | |  |  | 15% default | 8,069 | 8,492 |
|  | Default | 154 | 101 | 3% default | 280 | 188 |
|  | |  |  | 15% default | 1,365 | 942 |
|  | Death | 368 | 368 | 3% default | 592 | 592 |
|  | |  |  | 15% default | 566 | 566 |
| Completed and cured | | 9,098 | 9,163 | 3% default | 8,755 | 8,853 |
|  | |  |  | 15% default | 7,747 | 8,157 |
| Defaulted but cured | | 97 | 55 | 3% default | 174 | 104 |
|  | |  |  | 15% default | 742 | 434 |
| Total MDR treatment initiated | | 49 | 42 | 3% default | 10 | 5 |
|  | |  |  | 15% default | 6 | 4 |
| Total survival events (chronic TB) | | 50 | 43 | 3% default | 59 | 57 |
|  | |  |  | 15% default | 131 | 118 |
| Death if of chronic TB cases | | 138 | 146 | 3% default | 200 | 180 |
|  | |  |  | 15% default | 440 | 365 |
| Death during MDR treatment | | 2 | 3 | 3% default | 1 | 1 |
|  | |  |  | 15% default | 0 | 1 |
| Total deaths | | 525 | 522 | 3% default | 812 | 786 |
|  | |  |  | 15% default | 1,033 | 954 |

| Overall default | 1.5% | 1.0% | 3% default | 2.8% | 1.9% |
| --- | --- | --- | --- | --- | --- |
|  |  |  | 15% default | 13.7% | 9.4% |
| Relative reduction, default | ref | 34.4% | 3% default | ref | 32.9% |
|  |  |  | 15% default | ref | 31.0% |
| Absolute reduction, default | ref | 0.5% | 3% default | ref | 0.9% |
|  |  |  | 15% default | ref | 4.2% |
| Mortality during first-line treatment | 3.7% | 3.7% | 3% default | 5.9% | 5.9% |
|  |  |  | 15% default | 5.7% | 5.7% |
| Total mortality | 5.3% | 5.2% | 3% default | 8.1% | 7.9% |
|  |  |  | 15% default | 10.3% | 9.5% |
| Relative reduction, mortality | ref | 0.6% | 3% default | ref | 3.2% |
|  |  |  | 15% default | ref | 7.6% |
| Absolute reduction, mortality | ref | 0.0% | 3% default | ref | 0.3% |
|  |  |  | 15% default | ref | 0.8% |

1. Bangladesh

|  | | **Guidelines** | |  | **Current** | |
| --- | --- | --- | --- | --- | --- | --- |
|  |  | 6mo regimen | 4mo regimen |  | 6mo regimen | 4mo regimen |
| Initially detected as MDR patients | | 0 | 0 | 2% default | 0 | 0 |
|  | |  |  | 15% default | 0 | 0 |
| Started on first-line treatment | | 10,000 | 10,000 | 2% default | 10,000 | 10,000 |
|  | |  |  | 15% default | 10,000 | 10,000 |
|  | Complete | 9,562 | 9,621 | 2% default | 9,516 | 9,582 |
|  | |  |  | 15% default | 8,400 | 8,795 |
|  | Default | 155 | 96 | 2% default | 199 | 133 |
|  | |  |  | 15% default | 1,345 | 950 |
|  | Death | 283 | 283 | 2% default | 285 | 285 |
|  | |  |  | 15% default | 255 | 255 |
| Completed and cured | | 9,203 | 9,231 | 2% default | 9,161 | 9,200 |
|  | |  |  | 15% default | 8,072 | 8,446 |
| Defaulted but cured | | 96 | 52 | 2% default | 127 | 81 |
|  | |  |  | 15% default | 748 | 458 |
| Total MDR treatment initiated | | 51 | 51 | 2% default | 5 | 10 |
|  | |  |  | 15% default | 5 | 12 |
| Total survival events (chronic TB) | | 52 | 44 | 2% default | 51 | 55 |
|  | |  |  | 15% default | 148 | 125 |
| Death if of chronic TB cases | | 129 | 148 | 2% default | 176 | 167 |
|  | |  |  | 15% default | 427 | 377 |
| Death during MDR treatment | | 2 | 12 | 2% default | 0 | 4 |
|  | |  |  | 15% default | 0 | 3 |
| Total deaths | | 427 | 449 | 2% default | 477 | 463 |
|  | |  |  | 15% default | 702 | 649 |

| Overall default | 1.6% | 1.0% | 2% default | 2.0% | 1.3% |
| --- | --- | --- | --- | --- | --- |
|  |  |  | 15% default | 13.5% | 9.5% |
| Relative reduction, default | ref | 38.1% | 2% default | ref | 33.2% |
|  |  |  | 15% default | ref | 29.4% |
| Absolute reduction, default | ref | 0.6% | 2% default | ref | 0.7% |
|  |  |  | 15% default | ref | 4.0% |
| Mortality during first-line treatment | 2.8% | 2.8% | 2% default | 2.9% | 2.9% |
|  |  |  | 15% default | 2.6% | 2.6% |
| Total mortality | 4.3% | 4.5% | 2% default | 4.8% | 4.6% |
|  |  |  | 15% default | 7.0% | 6.5% |
| Relative reduction, mortality | ref | -5.2% | 2% default | ref | 2.9% |
|  |  |  | 15% default | ref | 7.5% |
| Absolute reduction, mortality | ref | -0.2% | 2% default | ref | 0.1% |
|  |  |  | 15% default | ref | 0.5% |

1. Brazil

|  | | **Guidelines** | |  | **Current** | |
| --- | --- | --- | --- | --- | --- | --- |
|  |  | 6mo regimen | 4mo regimen |  | 6mo regimen | 4mo regimen |
| Initially detected as MDR patients | | 316 | 316 | 21% default | 315 | 315 |
|  | |  |  | 50% default | 312 | 312 |
| Started on first-line treatment | | 9,684 | 9,684 | 21% default | 9,685 | 9,685 |
|  | |  |  | 50% default | 9,688 | 9,688 |
|  | Complete | 9,236 | 9,279 | 21% default | 7,465 | 7,967 |
|  | |  |  | 50% default | 5,241 | 6,276 |
|  | Default | 144 | 101 | 21% default | 1,789 | 1,287 |
|  | |  |  | 50% default | 4,009 | 2,974 |
|  | Death | 304 | 304 | 21% default | 431 | 431 |
|  | |  |  | 50% default | 438 | 438 |
| Completed and cured | | 8,926 | 9,010 | 21% default | 7,252 | 7,725 |
|  | |  |  | 50% default | 5,081 | 6,088 |
| Defaulted but cured | | 98 | 62 | 21% default | 1,111 | 714 |
|  | |  |  | 50% default | 2,394 | 1,593 |
| Total MDR treatment initiated | | 333 | 322 | 21% default | 151 | 149 |
|  | |  |  | 50% default | 143 | 144 |
| Total survival events (chronic TB) | | 61 | 49 | 21% default | 180 | 169 |
|  | |  |  | 50% default | 319 | 289 |
| Death if of chronic TB cases | | 168 | 158 | 21% default | 551 | 509 |
|  | |  |  | 50% default | 1,024 | 924 |
| Death during MDR treatment | | 31 | 31 | 21% default | 17 | 17 |
|  | |  |  | 50% default | 15 | 15 |
| Total deaths | | 505 | 496 | 21% default | 1,016 | 969 |
|  | |  |  | 50% default | 1,508 | 1,405 |

| Overall default | 1.5% | 1.0% | 21% default | 18.5% | 13.3% |
| --- | --- | --- | --- | --- | --- |
|  |  |  | 50% default | 41.4% | 30.7% |
| Relative reduction, default | ref | 29.9% | 21% default | ref | 28.1% |
|  |  |  | 50% default | ref | 25.8% |
| Absolute reduction, default | ref | 0.4% | 21% default | ref | 5.2% |
|  |  |  | 50% default | ref | 10.7% |
| Mortality during first-line treatment | 3.1% | 3.1% | 21% default | 4.5% | 4.5% |
|  |  |  | 50% default | 4.5% | 4.5% |
| Total mortality | 5.1% | 5.0% | 21% default | 10.2% | 9.7% |
|  |  |  | 50% default | 15.1% | 14.1% |
| Relative reduction, mortality | ref | 1.8% | 21% default | ref | 4.6% |
|  |  |  | 50% default | ref | 6.8% |
| Absolute reduction, mortality | ref | 0.1% | 21% default | ref | 0.5% |
|  |  |  | 50% default | ref | 1.0% |

**Figure S4. Cost-effectiveness planes by scenario (South Africa).**

**a) Guidelines scenario b) Current scenario**

**
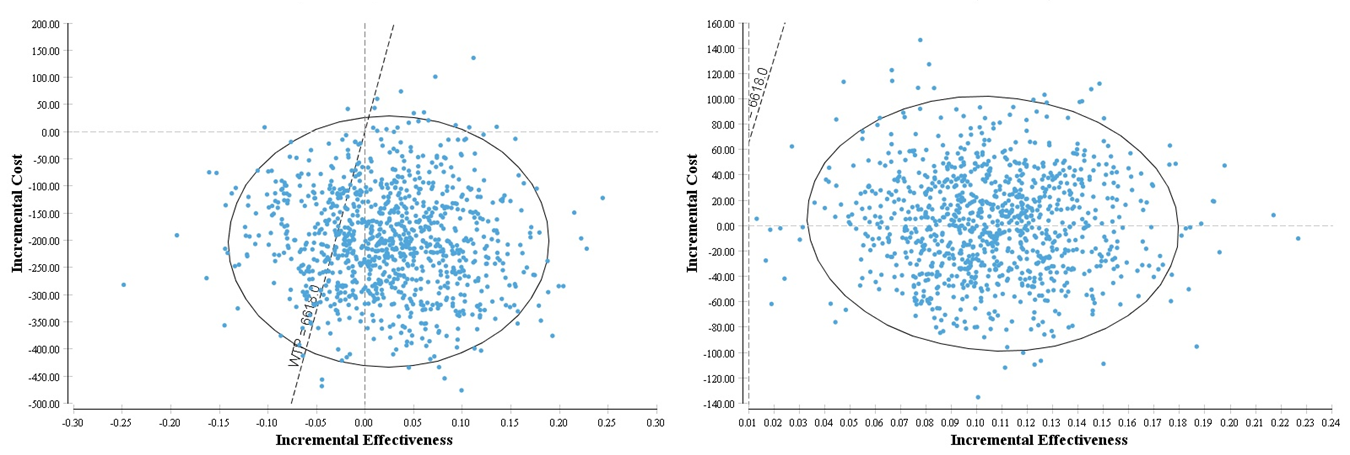
**

Incremental cost is represented in USD (2013) and incremental effectiveness is the number of DALYs averted. These are average per patient. WTP indicates the willingness to pay threshold (set at 1 gross domestic product). Each blue dot is the result of for one cohort of 10,000 new TB patients.

**Figure S5. Cost-effectiveness planes by scenario (Brazil).**

**a) Guidelines scenario b) Current scenario**

**
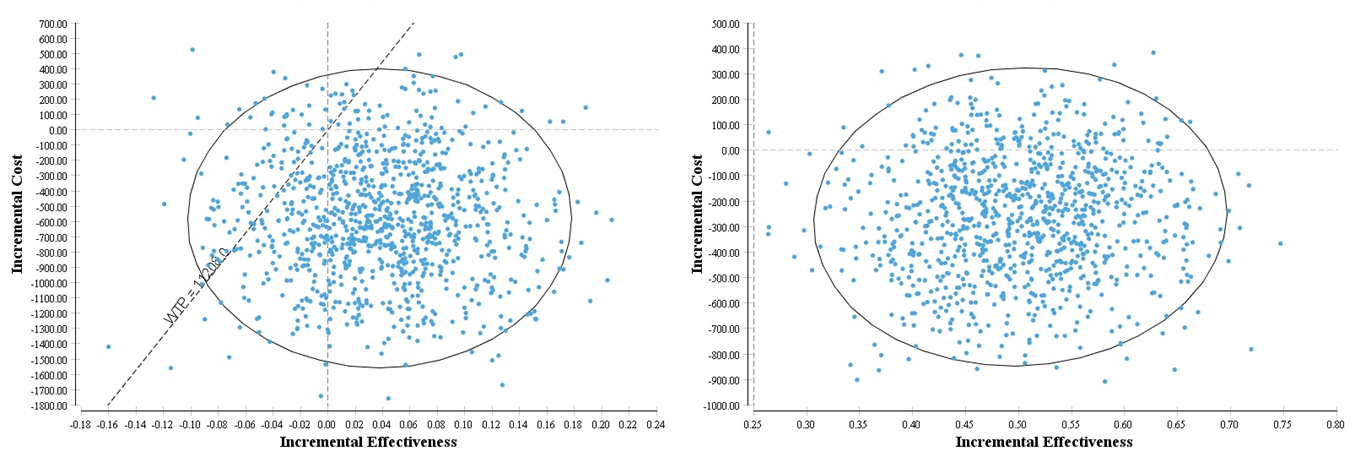
**

Incremental cost is represented in USD (2013) and incremental effectiveness is the number of DALYs averted. These are average per patient. WTP indicates the willingness to pay threshold (set at 1 gross domestic product). Each blue dot is the result of for one cohort of 10,000 new TB patients.

**Figure S6. Cost-effectiveness planes by scenario (Tanzania).**

**a) Guidelines scenario b) Current scenario**

**
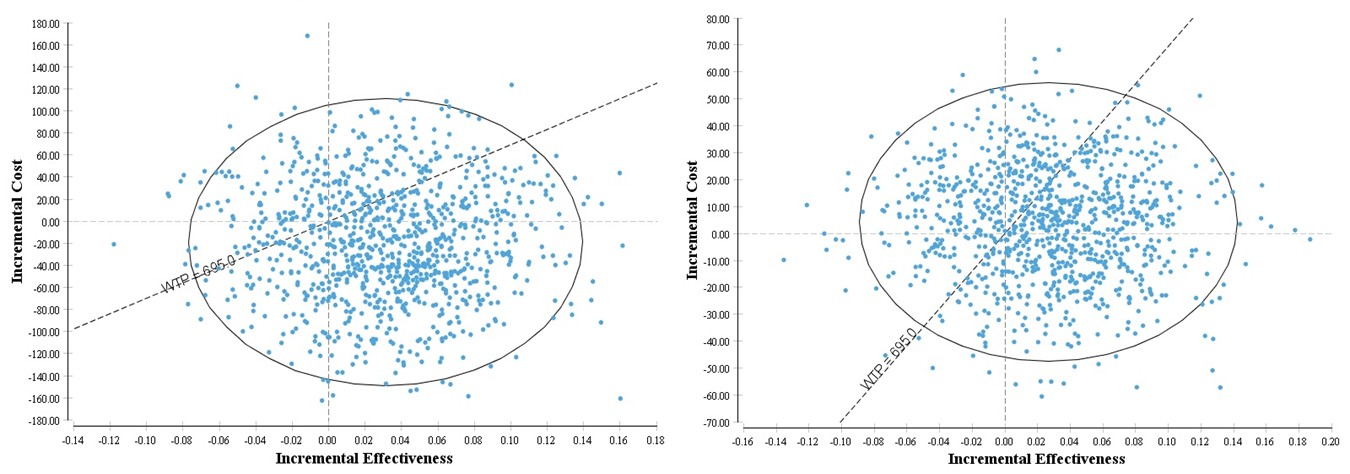
**

Incremental cost is represented in USD (2013) and incremental effectiveness is the number of DALYs averted. These are average per patient. WTP indicates the willingness to pay threshold (set at 1 gross domestic product). Each blue dot is the result of for one cohort of 10,000 new TB patients.

**Figure S7. Cost-effectiveness planes by scenario (Bangladesh).**

**a) Guidelines scenario b) Current scenario**

**
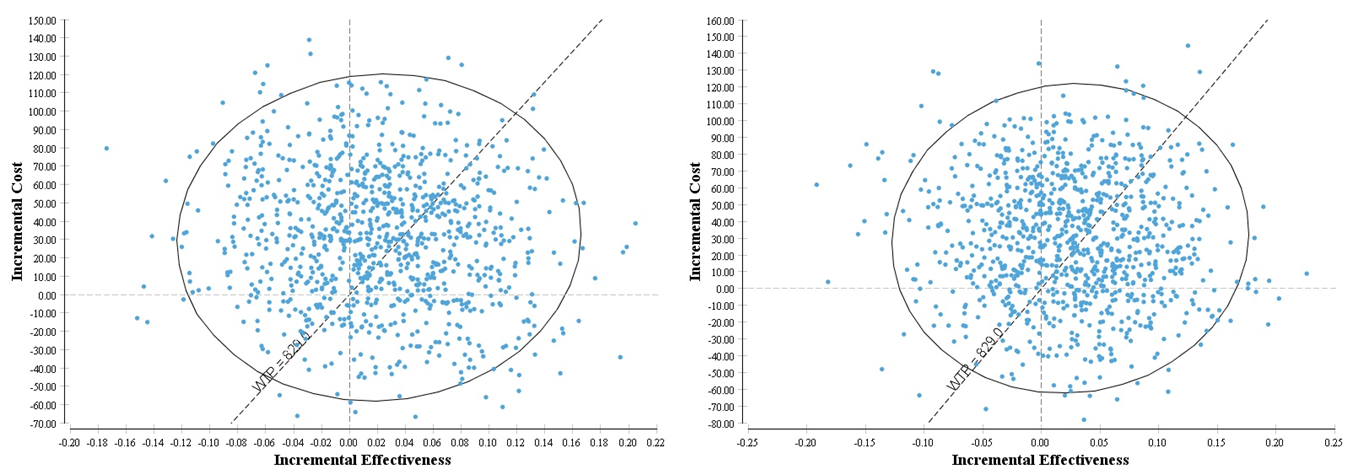
**

Incremental cost is represented in USD (2013) and incremental effectiveness is the number of DALYs averted. These are average per patient. WTP indicates the willingness to pay threshold (set at 1 gross domestic product). Each blue dot is the result of for one cohort of 10,000 new TB patients.

**Table S9. Threshold analysis by country and scenario.**

|  |  |  | **Guidelines** |  | **Current** |  |
| --- | --- | --- | --- | --- | --- | --- |
| **Country** | **ICER threshold** | | **average drug price per month** | **% runs > threshold (mean ICER)** | **average drug price per month** | **% runs > threshold (mean ICER)** |
| South Africa | 1/4xGDP | 1,655 | 94 | 24.5% (1,751.04) | 79 | 45.4% (1,682.09) |
|  | 1/2xGDP | 3,309 | 104 | 17.6% (3,326.82) | 128 | 45.0% (3,348.92) |
|  | 1xGDP | 6,618 | 125 | 15.0% (6,635.94) | 225 | 44.8% (6,648.56) |
| Brazil | 1/4xGDP | 2,802 | 221 | 30.3% (2,875.21) | 490 | 55.2% (2,876.05) |
|  | 1/2xGDP | 5,604 | 253 | 20.7% (5,623.96) | 858 | 52.5% (5,606.14) |
|  | 1xGDP | 11,208 | 319 | 16.0% (11,293.25) | 1,614 | 52.6% (11,214.71) |
| Tanzania | 1/4xGDP | 174 | 31 | 40.6% (321.19) | 31 | 31.1% (283.01) |
|  | 1/2xGDP | 348 | 32 | 33.4% (688.26) | 32 | 21.6% (418.24) |
|  | 1xGDP | 695 | 33 | 22.5% (1,055.33) | 35 | 15.4% (823.93) |
| Bangladesh | 1/4xGDP | 207 | 24 | 32.7% (300.65) | 24 | 34.0% (257.69) |
|  | 1/2xGDP | 415 | 25 | 22.7% (444.88) | 26 | 22.7% (517.00) |
|  | 1xGDP | 829 | 28 | 14.2% (877.57) | 29 | 14.9% (905.97) |

**Table S10. Detailed one-way sensitivity analysis (current scenario, drug price 1USD per day)**

|  |  |  | **6 months regime** | **4 months regimen** | **effect diff** | **6 months regime** | **4 months regimen** | **cost diff** | **ICER** |
| --- | --- | --- | --- | --- | --- | --- | --- | --- | --- |
|  |  |  | **DALY averted** | **DALY averted** |  | **TB-related cost, societal** | **TB-related cost, societal** |  |  |
| **South Africa** | | | | | | | | | |
|  | baseline |  | 8.26 | 8.37 | 0.11 | 831.35 | 832.84 | 1.50 | 13.62 |
| default rate during first-line treatment | high | 0.30 | 7.55 | 7.93 | 0.38 | 821.53 | 831.95 | 10.43 | 27.49 |
|  | low | 0.01 | 8.50 | 8.51 | 0.01 | 837.78 | 834.92 | -2.86 | CS |
| prevalence of MDR | high | 0.2 | 7.46 | 7.55 | 0.08 | 2,399.79 | 2,399.00 | -0.79 | CS |
|  | low | 0.001 | 8.34 | 8.45 | 0.11 | 687.76 | 689.26 | 1.50 | 13.70 |
| no partial cure if default (first-line treatment) | |  | 8.10 | 8.25 | 0.15 | 845.31 | 839.47 | -5.83 | CS |
| probability returning to care after default | high | 1 | 8.44 | 8.51 | 0.07 | 853.04 | 849.21 | -3.83 | CS |
|  | low | 0 | 8.16 | 8.29 | 0.12 | 816.84 | 819.77 | 2.92 | 23.54 |
| mortality during first-line treatment | high | Baseline x 2 | 7.84 | 7.94 | 0.10 | 812.13 | 814.94 | 2.81 | 27.73 |
|  | low | Baseline x 1/2 | 8.49 | 8.59 | 0.11 | 847.64 | 847.00 | -0.65 | CS |
| mortality after default, if no return to care | high | 1 | 8.26 | 8.37 | 0.11 | 831.35 | 832.84 | 1.50 | 13.62 |
|  | low | 0 | 8.26 | 8.37 | 0.11 | 831.35 | 832.84 | 1.50 | 13.62 |
| survival on ART |  | life expectancy | 10.25 | 10.38 | 0.13 | 831.35 | 832.84 | 1.50 | 11.14 |
| discount rate | high | 0.1 | 4.61 | 4.67 | 0.06 | 831.35 | 832.84 | 1.50 | 24.68 |
|  | low | 0 | 11.85 | 12.00 | 0.15 | 831.35 | 832.84 | 1.50 | 9.73 |
| **Brazil** | | | | | | | | | |
|  | baseline |  | 14.68 | 15.18 | 0.50 | 1,288.90 | 1,026.71 | -262.19 | CS |
| default rate during first-line treatment | high | 0.5 | 13.14 | 14.25 | 1.11 | 1,164.62 | 983.07 | -181.55 | CS |
|  | low | 0.01 | 15.87 | 15.89 | 0.03 | 1,381.38 | 1,062.45 | -318.92 | CS |
| prevalence of MDR | high | 0.2 | 12.92 | 13.33 | 0.41 | 1,492.81 | 1,276.28 | -216.53 | CS |
|  | low | 0.001 | 14.81 | 15.32 | 0.50 | 1,277.30 | 1,007.73 | -269.57 | CS |
| no partial cure if default (first-line treatment) | |  | 13.93 | 14.59 | 0.66 | 1,308.71 | 1,045.34 | -263.36 | CS |
| probability returning to care after default | high | 1 | 15.49 | 15.85 | 0.36 | 1,330.64 | 1,074.40 | -256.24 | CS |
|  | low | 0 | 14.29 | 14.85 | 0.57 | 1,273.38 | 1,016.09 | -257.28 | CS |
| mortality during first-line treatment | high | Baseline x 2 | 14.26 | 14.74 | 0.48 | 1,265.54 | 1,003.63 | -261.92 | CS |
|  | low | Baseline x 1/2 | 14.94 | 15.44 | 0.50 | 1,311.80 | 1,037.56 | -274.23 | CS |
| mortality after default, if no return to care | high | 1 | 14.68 | 15.18 | 0.50 | 1,288.90 | 1,026.71 | -262.19 | CS |
|  | low | 0 | 14.68 | 15.18 | 0.50 | 1,288.90 | 1,026.71 | -262.19 | CS |
| survival on ART |  | life expectancy | 15.52 | 16.05 | 0.52 | 1,288.90 | 1,026.71 | -262.19 | CS |
| discount rate | high | 0.1 | 4.83 | 4.98 | 0.15 | 1,288.90 | 1,026.71 | -262.19 | CS |
|  | low | 0 | 23.86 | 24.62 | 0.75 | 1,288.90 | 1,026.71 | -262.19 | CS |
| **Bangladesh** | | | | | | | | | |
|  | baseline |  | 16.17 | 16.20 | 0.02 | 856.48 | 886.32 | 29.84 | 1,220.93 |
| default rate during first-line treatment | high | 0.15 | 15.54 | 15.67 | 0.13 | 842.77 | 877.73 | 34.96 | 271.23 |
|  | low | 0.01 | 16.18 | 16.19 | 0.02 | 856.50 | 886.28 | 29.78 | 1,906.71 |
| prevalence of MDR | high | 0.2 | 14.66 | 14.69 | 0.03 | 983.19 | 916.77 | -66.42 | CS |
|  | low | 0.001 | 16.25 | 16.27 | 0.02 | 853.29 | 884.05 | 30.76 | 1,458.51 |
| no partial cure if default (first-line treatment) | |  | 16.04 | 16.12 | 0.08 | 856.59 | 886.82 | 30.23 | 363.89 |
| probability returning to care after default | high | 1 | 16.22 | 16.24 | 0.01 | 856.49 | 887.66 | 31.17 | 2,348.73 |
|  | low | 0 | 16.14 | 16.17 | 0.03 | 854.57 | 884.42 | 29.85 | 896.95 |
| mortality during first-line treatment | high | Baseline x 2 | 15.72 | 15.74 | 0.02 | 847.14 | 877.34 | 30.21 | 1,519.53 |
|  | low | Baseline x 1/2 | 16.35 | 16.38 | 0.03 | 859.05 | 889.28 | 30.23 | 967.00 |
| mortality after default, if no return to care | high | 1 | 16.17 | 16.20 | 0.02 | 856.48 | 886.32 | 29.84 | 1,220.93 |
|  | low | 0 | 16.17 | 16.20 | 0.02 | 856.48 | 886.32 | 29.84 | 1,220.93 |
| survival on ART |  | life expectancy | 16.29 | 16.32 | 0.03 | 856.48 | 886.32 | 29.84 | 961.33 |
| discount rate | high | 0.1 | 6.61 | 6.63 | 0.02 | 856.48 | 886.32 | 29.84 | 1,480.14 |
|  | low | 0 | 26.28 | 26.36 | 0.08 | 856.48 | 886.32 | 29.84 | 372.99 |
| **Tanzania** | | | | | | | | | |
|  | baseline |  | 12.97 | 13.00 | 0.03 | 598.19 | 602.48 | 4.29 | 161.22 |
| default rate during first-line treatment | high | 0.15 | 12.55 | 12.66 | 0.11 | 593.01 | 599.62 | 6.61 | 62.16 |
|  | low | 0.01 | 13.05 | 13.06 | 0.01 | 599.39 | 602.97 | 3.57 | 328.17 |
| prevalence of MDR | high | 0.2 | 11.74 | 11.78 | 0.03 | 610.89 | 615.52 | 4.63 | 143.46 |
|  | low | 0.001 | 13.06 | 13.09 | 0.03 | 597.84 | 601.88 | 4.04 | 152.45 |
| no partial cure if default (first-line treatment) | |  | 12.81 | 12.91 | 0.10 | 602.27 | 603.77 | 1.50 | 15.72 |
| probability returning to care after default | high | 1 | 13.07 | 13.08 | 0.01 | 604.41 | 607.23 | 2.82 | 375.51 |
|  | low | 0 | 12.91 | 12.95 | 0.04 | 595.06 | 599.36 | 4.30 | 116.01 |
| mortality during first-line treatment | high | Baseline x 2 | 12.51 | 12.53 | 0.02 | 582.24 | 587.99 | 5.75 | 302.26 |
|  | low | Baseline x 1/2 | 13.21 | 13.23 | 0.03 | 606.98 | 609.53 | 2.55 | 87.76 |
| mortality after default, if no return to care | high | 1 | 12.97 | 13.00 | 0.03 | 598.19 | 602.48 | 4.29 | 161.22 |
|  | low | 0 | 12.97 | 13.00 | 0.03 | 598.19 | 602.48 | 4.29 | 161.22 |
| survival on ART |  | life expectancy | 14.43 | 14.46 | 0.03 | 598.19 | 602.48 | 4.29 | 139.34 |
| discount rate | high | 0.1 | 6.12 | 6.14 | 0.01 | 598.19 | 602.48 | 4.29 | 324.75 |
|  | low | 0 | 20.48 | 20.53 | 0.05 | 598.19 | 602.48 | 4.29 | 92.73 |

MDR, multidrug resistant TB; ART, antiretroviral treatment; diff, difference; ICER, incremental cost effectiveness ratio; DALY, disability-adjusted life year.

# **References**

1. World Health Organization. Global tuberculosis report 2014. Geneva, Switzerland: World Health Organization; 2014.

2. Republic of South Africa Department of Health. Multi-drug resistant tuberculosis. A policy framework on decentralised and deinstitutionalised management for South Africa. 2011.

3. Republic of South Africa Department of Health. South African National Tuberculosis Management Guidelines [Internet]. 2009. Available from: http://familymedicine.ukzn.ac.za/Libraries/Guidelines_Protocols/TB_Guidelines_2009.sflb.ashx

4. Republic of South Africa Department of Health. Guidelines for Management of Drug-Resistant Tuberculosis in South Africa. [Internet]. 2010. Available from: http://www.tbonline.info/media/uploads/documents/mdr-tb_sa_2010.pdf

5. Republic of South Africa Department of Health. Changes in the ART guidelines [Internet]. 2012. Available from: http://www.tbonline.info/media/uploads/documents/changes_to_art_guidelines.pdf

6. The United Republic of Tanzania Ministry of Health and Social Welfare. Manual of the National Tuberculosis and Leprosy Programme in Tanzania [Internet]. 5th ed. 2006. Available from: http://www.who.int/hiv/pub/guidelines/tanzania_tb.pdf

7. The United Republic of Tanzania Ministry of Health and Social Welfare. National guidelines for the management of HIV and AIDS [Internet]. 4th ed. 2012. Available from: http://www.nacp.go.tz/documents/nationalguideline42012.pdf

8. National Tuberculosis Control Programme Directorate General of Health Services Ministry of Health and Family Welfare Dhaka, Bangladesh. National Guidelines and Operational Manual for Tuberculosis Control [Internet]. 4th ed. 2011. Available from: http://www.scribd.com/doc/57371572/Bangladesh-National-Guidelines-and-Operational-Manual-for-Tuberculosis-Control

9. National AIDS /STD Program, Directorate General of Health Services, Ministry of Health and Family Welfare. National guidelines of antiretroviral therapy Bangladesh [Internet]. Dhaka, Bangladesh; 2011. Available from: http://ban.searo.who.int/LinkFiles/Publication_National_ART_Guide_August_2011_Bangladesh.pdf

10. Ministério da Saúde, Secretaria de Vigilância em Saúde, Departamento de Vigilância Epidemiológica. Manual de recomendações para o controle da tuberculose no Brasil [Internet]. Brasilia: Ministério da Saúde; 2011. Available from: http://portal.saude.gov.br/portal/arquivos/pdf/manual_de_recomendacoes_tb.pdf

11. Ministério da Saúde, Brazil. Recomendações de terapia antirretroviral para adult os vivendo com HIV/aids no Brasil – 2012 [Internet]. Available from: http://www.aids.gov.br/sites/default/files/anexos/publicacao/2012/52140/consenso_adulto2012_principais_mudancas_pdf_11946.pdf

12. Vassall A, Siapka M, Foster N, Fielding K, McCarthy K, Shillington L, et al. Xpert MTB/RIF scale-up in South Africa: impact on system-wide resource use and cost. In preparation.

13. Foster N, Vassall A, Cleary S, Cunnama L, Churchyard GJ, Sinanovic E. The economic burden of TB diagnosis and treatment in South Africa. Social Science and Medicine. 2015;130:e27.

14. Gospodarevskaya E, Tulloch O, Bunga C, Ferdous S, Jonas A, Islam S, et al. Patient costs during tuberculosis treatment in Bangladesh and Tanzania: the potential of shorter regimens. Int J Tuberc Lung Dis. 2014 Jul;18(7):810–7.

15. Zwerling A, Ferdous S, Basher AK, Islam S, Islam MA, Gomez GB, et al. Delivering first line TB treatment in Bangladesh: Facility costs and community health workers. In preparation.

16. Trajman A, Lisboa Bastos M, Belo M, Calaça J, Gaspar J, Martins dos Santos C, et al. Shortened first-line TB treatment in Brazil: potential cost savings for patients and health services. In preparation.

17. Wandwalo E, Robberstad B, Morkve O. Cost and cost-effectiveness of community based and health facility based directly observed treatment of tuberculosis in Dar es Salaam, Tanzania. Cost Eff Resour Alloc. 2005 Jul 14;3:6.

18. Rosen S, Long L, Sanne I. The outcomes and outpatient costs of different models of antiretroviral treatment delivery in South Africa. Trop Med Int Health. 2008 Aug;13(8):1005–15.

19. Long L, Fox M, Sanne I, Rosen S. The high cost of second-line antiretroviral therapy for HIV/AIDS in South Africa. AIDS. 2010 Mar 27;24(6):915–9.

20. Menzies NA, Berruti AA, Berzon R, Filler S, Ferris R, Ellerbrock TV, et al. The cost of providing comprehensive HIV treatment in PEPFAR-supported programs. AIDS. 2011 Sep 10;25(14):1753–60.

21. Bratt JH, Torpey K, Kabaso M, Gondwe Y. Costs of HIV/AIDS outpatient services delivered through Zambian public health facilities. Trop Med Int Health. 2011 Jan;16(1):110–8.

22. Chandrashekar S, Guinness L, Pickles M, Shetty GY, Alary M, Vickerman P, et al. The costs of scaling up HIV prevention for high risk groups: lessons learned from the Avahan Programme in India. PLoS ONE. 2014;9(9):e106582.

23. Siapka M, Remme M, Obure CD, Maier CB, Dehne KL, Vassall A. Is there scope for cost savings and efficiency gains in HIV services? A systematic review of the evidence from low- and middle-income countries. Bull World Health Organ. 2014 Jul 1;92(7):499–511AD.

24. Rosen S, Ketlhapile M, Sanne I, DeSilva MB. Cost to patients of obtaining treatment for HIV/AIDS in South Africa. S Afr Med J. 2007 Jul;97(7):524–9.

25. Boehme CC, Nicol MP, Nabeta P, Michael JS, Gotuzzo E, Tahirli R, et al. Feasibility, diagnostic accuracy, and effectiveness of decentralised use of the Xpert MTB/RIF test for diagnosis of tuberculosis and multidrug resistance: a multicentre implementation study. Lancet. 2011 Apr 30;377(9776):1495–505.

26. Prado TN do, Caus AL, Marques M, Maciel EL, Golub JE, Miranda AE. Epidemiological profile of adult patients with tuberculosis and AIDS in the state of Espírito Santo, Brazil: cross-referencing tuberculosis and AIDS databases. J Bras Pneumol. 2011 Feb;37(1):93–9.

27. Zaman K, Hossain S, Banu S, Quaiyum P, Barua PC, Hamid Salim MA, et al. Prevalence of smear-positive tuberculosis in persons aged ⩾15 years in Bangladesh: results from a national survey, 2007–2009. Epidemiology and Infection. 2012;140:1018–27.

28. Mahy M, Stover J, Stanecki K, Stoneburner R, Tassie J. Estimating the impact of antiretroviral therapy: regional and global estimates of life-years gained among adults. Sexually Transmitted Infections. 2010;86(Suppl 2):ii67–71.

29. Donnell D, Baeten J, Kiarie J, Thomas K, Stevens W, et al. Heterosexual HIV-1 transmission after initiation of antiretroviral therapy: a prospective cohort analysis. Lancet. 2010;375:2092–8.

30. Hollingsworth T, Anderson R, Fraser C. HIV-1 transmission, by stage of infection. J Infectious Diseases. 2008;198:687–93.

31. Murray CJL, Ezzati M, Flaxman AD, Lim S, Lozano R, Michaud C, et al. GBD 2010: design, definitions, and metrics. Lancet. 2012 Dec 15;380(9859):2063–6.
