# Supplementary material for: Cost and cost-effectiveness of tuberculosis treatment shortening: a model-based analysis
Source: BMC Infect Dis. 2016 Dec 1;16:726. doi: 10.1186/s12879-016-2064-3 (PMC5131398; doi:10.1186/s12879-016-2064-3)
Supplement: Additional file 2: — CHEERS checklist and technical appendix including additional results. (DOCX 16 kb) [file 12879_2016_2064_MOESM2_ESM.docx]

**CHEERS Checklist**

**Items to include when reporting economic evaluations of health interventions**

| **Section/item** | **Item**  **No** | **Recommendation** | **Reported**  **on page No** |
| --- | --- | --- | --- |
|  |  |  |  |
| **Title and abstract** |  |  |  |
| Title | 1 | Identify the study as an economic evaluation or use more  specific terms such as “cost-effectiveness analysis”, and  describe the interventions compared. | 1 |
| Abstract | 2 | Provide a structured summary of objectives, perspective,  setting, methods (including study design and inputs), results  (including base case and uncertainty analyses), and  conclusions. | 2 |
| **Introduction** |  |  |  |
| Background and  objectives | 3 | Provide an explicit statement of the broader context for the study. |  |
|  |  | Present the study question and its relevance for health policy or practice decisions. | 4 |
| **Methods** |  |  |  |
| Target population and subgroups | 4 | Describe characteristics of the base case population and  subgroups analysed, including why they were chosen. | 4 |
| Setting and location | 5 | State relevant aspects of the system(s) in which the decision(s) need(s) to be made. | 4 |
| Study perspective | 6 | Describe the perspective of the study and relate this to the  costs being evaluated. | 8 |
| Comparators | 7 | Describe the interventions or strategies being compared and  state why they were chosen. | 8 |
| Time horizon | 8 | State the time horizon(s) over which costs and consequences  are being evaluated and say why appropriate. | 11 |
| Discount rate | 9 | Report the choice of discount rate(s) used for costs and outcomes and say why appropriate | 11 |
| Choice of health  outcomes | 10 | Describe what outcomes were used as the measure(s) of  benefit in the evaluation and their relevance for the type of  analysis performed. | 4 |
| Measurement of effectiveness | 11a | Single study-based estimates: Describe fully the design  features of the single effectiveness study and why the single  study was a sufficient source of clinical effectiveness data. | n/a |
|  | 11b | Synthesis-based estimates: Describe fully the methods used for identification of included studies and synthesis of clinical  effectiveness data. | 5-7; suppl1 |
| Measurement and valuation of preference based outcomes | 12 | If applicable, describe the population and methods used to  elicit preferences for outcomes. | n/a |
| Estimating resources  and costs | 13a | Single study-based economic evaluation: Describe approaches used to estimate resource use associated with the alternative interventions. Describe primary or secondary research methods for valuing each resource item in terms of its unit cost. Describe any adjustments made to approximate to opportunity costs. | n/a |
|  | 13b | Model-based economic evaluation: Describe approaches and  data sources used to estimate resource use associated with  model health states. Describe primary or secondary research  methods for valuing each resource item in terms of its unit  cost. Describe any adjustments made to approximate to  opportunity costs. | 8-10;suppl1 |
| Currency, price date, and conversion | 14 | Report the dates of the estimated resource quantities and unit costs. Describe methods for adjusting estimated unit costs to the year of reported costs if necessary. Describe methods for converting costs into a common currency base and the exchange rate. | 8 |
| Choice of model | 15 | Describe and give reasons for the specific type of decision analytical model used. Providing a figure to show model structure is strongly recommended. | 4;suppl1 |
| Assumptions | 16 | Describe all structural or other assumptions underpinning the decision-analytical model. | 4-7 |
| Analytical methods | 17 | Describe all analytical methods supporting the evaluation. This could include methods for dealing with skewed, missing, or censored data; extrapolation methods; methods for pooling data; approaches to validate or make adjustments (such as half cycle corrections) to a model; and methods for handling population heterogeneity and uncertainty. | 10-11 |
| **Results** |  |  |  |
| Study parameters | 18 | Report the values, ranges, references, and, if used, probability distributions for all parameters. Report reasons or sources for distributions used to represent uncertainty where appropriate. Providing a table to show the input values is strongly recommended. | 4-11 |
| Incremental costs and outcomes | 19 | For each intervention, report mean values for the main  categories of estimated costs and outcomes of interest, as well as mean differences between the comparator groups. If  applicable, report incremental cost-effectiveness ratios. | 12-13; suppl1 |
| Characterising uncertainty | 20a | Single study-based economic evaluation: Describe the effects  of sampling uncertainty for the estimated incremental cost and incremental effectiveness parameters, together with the impact of methodological assumptions (such as discount rate, study perspective). | n/a |
|  | 20b | Model-based economic evaluation: Describe the effects on the results of uncertainty for all input parameters, and uncertainty related to the structure of the model and assumptions. | 13 |
| Characterising heterogeneity | 21 | If applicable, report differences in costs, outcomes, or cost-effectiveness that can be explained by variations between  subgroups of patients with different baseline characteristics or other observed variability in effects that are not reducible by more information. | n/a |
| **Discussion** |  |  |  |
| Study findings, limitations, generalisability, and current knowledge | 22 | Summarise key study findings and describe how they support  the conclusions reached. Discuss limitations and the  generalisability of the findings and how the findings fit with  current knowledge. | 13-14 |
| Other |  |  |  |
| Source of funding | 23 | Describe how the study was funded and the role of the funder in the identification, design, conduct, and reporting of the analysis. Describe other non-monetary sources of support. | 15 |
| Conflicts of interest | 24 | Describe any potential for conflict of interest of study contributors in accordance with journal policy. In the absence of a journal policy, we recommend authors comply with International Committee of Medical Journal Editors  recommendations. | 15 |
